# Supplementary material for: Natural Appetite Control: Consumer Perception of Food-Based Appetite Regulating Aromas
Source: Nutrients. 2023 Jun 30;15(13):2996. doi: 10.3390/nu15132996 (PMC10347076; doi:10.3390/nu15132996)
Supplement: Supplementary file 1 [file nutrients-15-02996-s001.zip › Supplementary materials File 1 - Questionnaire.pdf]

Intemperance in food consumption, results in excessive energy supply, which contributes to the obesity pandemic. Appetite, feelings of hunger and satiety, and food intake by humans are controlled by sensory, cognitive, hormonal and metabolism. The senses of taste and smell play a key role in the sensory impact of on the selection and consumption of food products.

Dear Participants,

we kindly request people over 60 years old or overweight/obese to complete the following survey and the Simplified Appetite Assessment Questionnaire (SNAQ). This questionnaire was created for research work related to a grant under the "Lider" program of the National Center for Research and Development (Poland), which aims to create a new generation of appetite regulators with a natural and safe to consumer odor. The survey is completely anonymous and confidential, so please complete it independently and conscientiously filling it out. Thank you in advance for your time and honest answers.

Survey information:

Please circle the one answer that best describes your correct answer (or several answers - in case of multiple choice questions). Questions 1 through 17, 24 and 52-53 are single-choice questions, while 18-23, 25-51 are multiple-choice. Please note that in questions 36 through 51 you are asked to provide only two answers.

I. Metrics

1. Gender (biological)
    a. Female
    b. Male
2. Year of birth: …..
3. Education
    a. Primary
    b. Secondary
    c. Vocational
    d. Higher
4. Place of residence
    a. City of up to 50,000 inhabitants
    b. City up to 100 thousand residents
    c. City of up to 250 thousand residents
    d. City of more than 250 thousand residents
    e. Village
5. The province in which you currently live:
    a. dolnośląskie
    b. kujawsko-pomorskie
    c. lubelskie
    d. lubuskie
    e. łódzkie
    f. małopolskie
    g. mazowieckie
    h. opolskie
    i. podkarpackie
    j. podlaskie
    k. pomorskie
    l. śląskie
    m. świętokrzyskie
    n. warmińsko-mazurskie
    o. wielkopolskie
    p. zachodniopomorskie
    q. I live temporarily or permanently outside of Poland

6. What is your body weight in kg?

...

7. What is your height in cm?

...

8. What is your waist circumference in cm?

...

(If you don't know, please indicate which waist circumference you choose when buying clothes? If it is possible, please measure your waist circumference with a tailor's centimeter).

9. Please specify what your figure looks like:

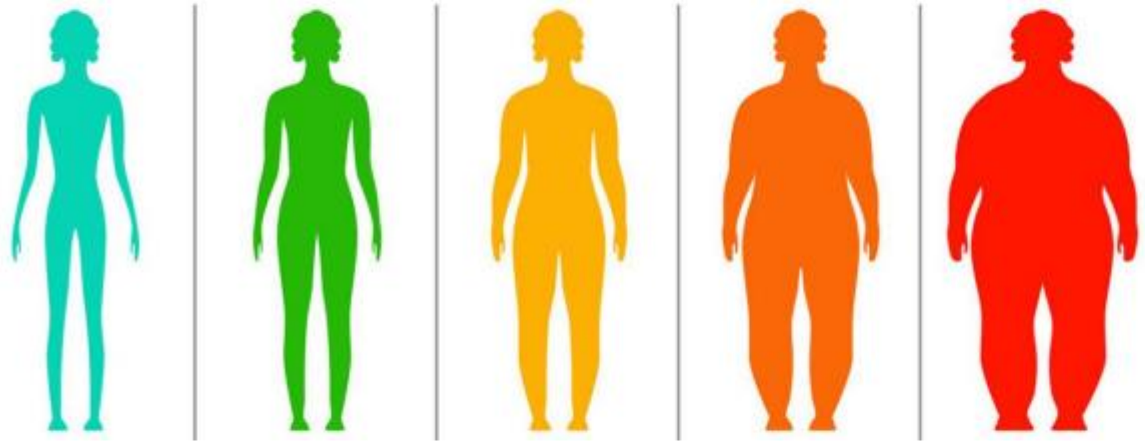

1 2 3 4 5

II. Simplified Nutronal Appetite Questionnaire (SNAQ).

1. My appetite is:

a. very weak
b. weak
c. moderate
d. good
e. very good

2. When I eat:

a. I feel full after eating a few bites
b. I feel full after eating 1/3 of a meal
c. I feel full after eating more than half of a meal
d. I feel full after eating most of the meal
e. I rarely feel fullness after eating a meal

3. Food tastes:

a. very bad

NCBR Narodowe Centrum Badań i Rozwoju

„Nowej generacji środki regulujące apetyt – użyteczne w opiece długoterminowej oraz kontroli masy ciała" Akronim RANG.e
Projekt finansowany przez Narodowe Centrum Badań i Rozwoju
Numer umowy: LIDER/48/0191/L-11/19/NCBR/2020

LIDER

b. bad

c. moderately

d. good

e. very good

4. I normally eat:

a. Less than one meal a day

b. One meal during the day

c. two meals during the day

d. three meals during the day

e. more than three meals in a day

III. Questionnaire

5. After drinking a glass of bitter black tea, do you feel satiety

(suppression of hunger)?

a. yes

b. moderately

c. no

d. I do not consume this product

6. After drinking a glass of black tea sweetened with 1 teaspoon of white sugar, do you do you feel satiety (suppression of hunger)?

a. yes

b. moderately

c. no

d. I do not consume this product

7. After drinking a glass of black tea with lemon juice, sweetened with 1 teaspoon of white sugar, do you feel satiety (suppression of hunger)?

a. yes

b. moderately

c. no

d. I do not consume this product

8. After drinking a glass of black tea sweetened with 1 teaspoon of white sugar (or other sweetener) do you feel hungry?

a. yes

b. moderately

c. no

d. I do not consume this product

NCBR Narodowe Centrum Badań i Rozwoju

„Nowej generacji środki regulujące apetyt – użyteczne w opiece długoterminowej oraz kontroli masy ciała" Akronim RANG.e
Projekt finansowany przez Narodowe Centrum Badań i Rozwoju
Numer umowy: LIDER/48/0191/L-11/19/NCBR/2020

LIDER

9. After drinking a glass of green tea, do you feel satiety (suppression of hunger)?

a. yes

b. moderately

c. no

d. I do not consume this product

10. Do you feel satiety (suppression of hunger) after drinking a glass of yerba mate?

a. yes

b. moderately

c. no

d. I do not consume this product

11. After drinking a cup of coffee (without added milk and/or sugar), do you feel hunger?

a. yes

b. moderately

c. no

d. I do not consume this product

12. After eating a dish containing raw chili peppers, do you feel satiety?

a. yes

b. moderately

c. no

d. I do not consume this product

13. After eating a product or food containing powdered chili peppers, do you do you feel satiety?

a. yes

b. moderately

c. no

d. I do not consume this product

14. Do products containing vanilla flavor increase your appetite? If yes then which of the following products have this effect?

a. no, it does not increase appetite

b. vanilla flavored tea

c. vanilla pudding

d. cake with the addition of vanilla oil

e. vanilla ice cream

15. What does the consumption of a product/food with added nutmeg trigger in you?

NCBR Narodowe Centrum Badań i Rozwoju

„Nowej generacji środki regulujące apetyt – użyteczne w opiece długoterminowej oraz kontroli masy ciała" Akronim RANG.e

Projekt finansowany przez Narodowe Centrum Badań i Rozwoju
Numer umowy: LIDER/48/0191/L-11/19/NCBR/2020

LIDER

a. definitely increases appetite
b. increases appetite
c. has no effect on appetite
d. reduces appetite
e. definitely decreases appetite

16. What does consuming a clove-scented product or food with the addition of cloves?
a. definitely increases appetite
b. increases appetite
c. has no effect on appetite
d. decreases appetite
e. definitely decreases appetite

17. What does consuming a cinnamon-scented product cause you to eat food with added cinnamon?
a. definitely increases appetite
b. increases appetite
c. has no effect on appetite
d. decreases appetite
e. definitely decreases appetite

18. After exposure to the smell of bananas, do you have an urge to eat any of the of the following products?
a. no, I don't feel like eating anything
b. bananas
c. milk chocolate
d. dark chocolate
e. cakes and cookies
f. vanilla yogurt
g. natural yogurt
h. jam
i. crackers
j. sticks
k. chips

19. After exposure to the smell of watermelon, do you feel like eating any of the of the following products?
a. no, I don't feel like eating anything
b. bananas
c. milk chocolate
d. dark chocolate

e. cakes and cookies

f. vanilla yogurt

g. natural yogurt

h. jam

i. crackers

j. sticks

k. chips

20. After exposure to the smell of vanilla/chocolate pudding, do you feel like to eat any of the following products?

a. no, I don't feel like eating anything

b. bananas

c. milk chocolate

d. dark chocolate

e. cakes and cookies

f. vanilla yogurt

g. natural yogurt

h. jam

i. crackers

j. sticks

k. chips

21. After exposure to the smell of raw vegetables, do you feel like eating them? If yes, please let me know what kind of vegetables they are?

a. yes, I feel like consuming brassica vegetables (broccoli, cauliflower, kale, kohlrabi, white cabbage, savoy cabbage, Chinese cabbage) after smelling him/her/they

b. yes, I feel like consuming onion vegetables (onions, garlic, chives) after smelling it/ them

smelling it/him/her/their

c. yes, I feel like consuming leafy vegetables (radicchio, parsley, lamb's lettuce, arugula, lettuce, spinach) after smelling it/him/her/their

d. yes, I feel like consuming root vegetables (beet, carrot, parsley) after smelling it/him/her/their

e. yes, I feel like consuming solanaceous vegetables (bell pepper, tomato) after smelling it/ them him/her/them

f. yes, I feel like consuming a green cucumber after smelling it/ them

g. yes, I feel like consuming pumpkin after smelling it/ them

h. yes, I feel like consuming rutabaga, turnip, radish, radish after smelling it/they

i. yes, I feel like consuming mushrooms after smelling them

j. no, I do not feel like it

22. After exposure to the smell of raw fruits, do you feel like eating them? If yes, please let me know what kind of fruit it is?

   a. yes, I feel like consuming citrus fruits (lemon, orange, tangerine, pamelo, grapefruit) after smelling it/they

   b. yes, I feel like consuming exotic fruits (lychee, pomegranate, passion fruit, papaya) after smelling it/ them

   c. yes, I feel like consuming figs/dates after smelling them

   d. yes, I feel like consuming an apple/ pear after smelling it/ them

   e. yes, I feel like consuming cherries/cherries after smelling them

   f. yes, I feel like consuming a peach/ apricot/ nectarine after smelling it/ them

   g. yes, I feel like consuming strawberries/strawberries/raspberries/blueberries/blueberries/ blackberries after smelling them

   h. yes, I feel like consuming bananas after smelling them

   i. no, I do not feel like it

23. After being exposed to the smell of cooked vegetables, do you feel like eating them? If yes, please let me know what kind of vegetables they are?

   a. yes, I feel like consuming brassica vegetables (broccoli, cauliflower, kale, kohlrabi, white cabbage, savoy cabbage) after smelling it/ them

   b. yes, I feel like consuming onion vegetables (onions, garlic, chives) after smelling it/ them smelling it/him/her/their

   c. yes, I feel like consuming leafy vegetables (spinach) after smelling it/her/their

   d. yes, I feel like consuming root vegetables (beet, carrot, parsley) after smelling it/her/their smelling it/him/her/their

   e. yes, I feel like consuming solanaceous vegetables (bell pepper, tomato) after smelling it/ them him/her/them

   f. yes, I feel like consuming potatoes after smelling them

   g. yes, I feel like consuming pumpkins after smelling them

   h. yes, I feel like consuming mushrooms after smelling them

   i. no, I do not feel like it

24. After exposure to the smell of fresh tomatoes, do you feel like consuming

tomato soup?

   a. yes

   b. no

25. After passing by a pastry shop/bakery where the aroma of bread spreads do you feel like consuming any of the following products?

   a. rye bread

   b. graham bread

c. wheat bread

d. rye roll

e. graham bread

f. wheat roll

g. corn roll

h. sweet roll

i. other products (what kind?)

j. I don't feel like consuming anything

26. After eating foods with an intense sweet taste (milk chocolate, bar with chocolate, ...) do you feel like consuming any of the following products?

a. pork chop

b. beef stew

c. chicken dish

d. fried chicken

e. grilled chicken

f. grits

g. rice

h. potatoes

i. natural yogurt

j. flavored yogurt (strawberry, berry, etc.)

k. cream cheese

l. chocolate

m. cookies

n. crackers

o. sticks

p. chips

q. fast food (e.g., hamburger, fries, hot dog, pizza)

r. other product/food (what kind?)

s. don't feel like consuming anything

27.Aafter eating foods of umami flavor intensity (with added MSG monosodium, e.g., Chinese soup, hot cup, bouillon from a cube) do you have an urge to to consume any of the following products?

a. pork chop

b. beef stew

c. chicken dish

d. fried chicken

e. grilled chicken
f. grits
g. rice
h. potatoes
i. natural yogurt
j. flavored yogurt (strawberry, berry, etc.)
k. cream cheese
l. chocolate
m. cookies
n. crackers
o. sticks
p. chips
q. fast food (e.g., hamburger, fries, hot dog, pizza)
r. other product/food (what kind?)
s. I don't feel like consuming anything

28. After eating food of salty flavor intensity, do you feel like to consume any of the following foods?

a. pork chop
b. beef stew
c. chicken dish
d. fried chicken
e. grilled chicken
f. grits
g. rice
h. potatoes
i. natural yogurt
j. flavored yogurt (strawberry, berry, etc.)
k. cream cheese
l. chocolate
m. cookies
n. crackers
o. sticks
p. chips
q. fast food (e.g., hamburger, fries, hot dog, pizza)
r. other product/food (what kind?)

s. I don't feel like consuming anything

29. After eating foods with oil (chips, fat-fried meats) do you do you feel like consuming any of the following foods?

a. pork chop

b. beef stew

c. chicken dish

d. fried chicken

e. grilled chicken

f. grits

g. rice

h. potatoes

i. natural yogurt

j. flavored yogurt (strawberry, berry, etc.)

k. cream cheese

l. chocolate

m. cookies

n. crackers

o. sticks

p. chips

q. fast food (e.g., hamburger, fries, hot dog, pizza)

r. other product/food (what kind?)

s. I don't feel like consuming anything

30. What are your feelings after exposure to the smell of green lettuce?

a. the appetite for sweets is reduced

b. the appetite for spicy products (with the addition of red chili peppers, for example) is reduced

c. the appetite for flour products (pasta, noodles, pancakes) is reduced

d. the appetite for poultry and/or beef, pork is reduced

e. the appetite for dairy products is reduced

f. the appetite for sweets is increased

g. the appetite for spicy products (with the addition of red chili peppers, for example) increases

h. the appetite for flour products (pasta, noodles, pancakes) increases

i. the appetite for poultry and/or beef, pork increases

j. the appetite for dairy products increases

k. the appetite for another product (which one?) is decreased

NCBR Narodowe Centrum Badań i Rozwoju

„Nowej generacji środki regulujące apetyt – użyteczne w opiece długoterminowej oraz kontroli masy ciała" Akronim RANG.e

Projekt finansowany przez Narodowe Centrum Badań i Rozwoju
Numer umowy: LIDER/48/0191/L-11/19/NCBR/2020

LIDER

l. appetite for another product is increased (what?)

m. does not apply (no increase/decrease in appetite)

31. What are your feelings after exposure to the smell of dark chocolate?

a. the appetite for sweets is reduced

b. the appetite for spicy products (with the addition of red chili peppers, for example) is reduced

c. the appetite for flour products (pasta, noodles, pancakes) is reduced

d. the appetite for poultry and/or beef, pork is reduced

e. the appetite for dairy products is reduced

f. the appetite for sweets is increased

g. the appetite for spicy products (with the addition of red chili peppers, for example) increases

h. the appetite for flour products (pasta, noodles, pancakes) increases

i. the appetite for poultry and/or beef, pork increases

j. the appetite for dairy products increases

k. the appetite for another product (which one?) is decreased

l. appetite for another product is increased (what?)

m. not applicable (no increase/decrease in appetite)

32. What are your feelings after exposure to the smell of yellow cheese?

a. the appetite for sweets is reduced

b. the appetite for spicy products (with the addition of red chili peppers, for example) is reduced

c. the appetite for flour products (pasta, noodles, pancakes) is reduced

d. the appetite for poultry and/or beef, pork is reduced

e. the appetite for dairy products is reduced

f. the appetite for sweets is increased

g. the appetite for spicy products (with the addition of red chili peppers, for example) increases

h. the appetite for flour products (pasta, noodles, pancakes) increases

i. the appetite for poultry and/or beef, pork increases

j. the appetite for dairy products increases

k. the appetite for another product (which one?) is decreased

l. appetite for another product is increased (what?)

m. not applicable (no increase/decrease in appetite)

33. What are your feelings after exposure to the smell of croquet with cabbage and mushrooms?

a. the appetite for sweets is reduced

b. the appetite for spicy products (with the addition of red chili peppers, for example) is reduced

c. the appetite for flour products (pasta, noodles, pancakes) is reduced

d. the appetite for poultry and/or beef, pork is reduced

e. the appetite for dairy products is reduced

f. the appetite for sweets is increased

g. the appetite for spicy products (with the addition of red chili peppers, for example) increases

h. the appetite for flour products (pasta, noodles, pancakes) increases

i. the appetite for poultry and/or beef, pork increases

j. the appetite for dairy products increases

k. the appetite for another product (which one?) is decreased

l. appetite for another product is increased (what?)

m. not applicable (no increase/decrease in appetite)

34. Odors of which products make you feel unsafe?

a. coffee

b. black tea

c. dark chocolate

d. yellow cheese

e. blue cheese

f. boiled egg

g. fried salmon

h. oatmeal on milk

i. homogenized vanilla cheese

j. chocolate ice cream

k. vanilla ice cream

l. fruit sorbet

m. another product (what kind?)

n. I do not have this feeling after eating any product

35. The smells of which products make you feel blissful?

a. coffee

b. black tea

c. dark chocolate

d. yellow cheese

e. blue cheese

f. boiled egg

g. fried salmon

h. oatmeal on milk

i. homogenized vanilla cheese

j. chocolate ice cream

k. vanilla ice cream

l. fruit sorbet

m. another product (what kind?)

n. I do not have this feeling after eating any product

36. Please mark two of the following foods that you find most appetizing.

a. vegetable salad (consisting of cucumber, tomato, lettuce, dried basil and olive oil)

b. beef stew with buckwheat groats

c. letcho with chicken and white rice

d. sour cucumber soup

e. tomato soup with pasta

f. pancakes with cottage cheese and jam

g. dumplings with cheese and potatoes

h. croquette with cabbage and mushrooms

i. cod fried in butter with garlic and dill

37. Please mark two of the following dishes that you find least appetizing.

a. vegetable salad (consisting of cucumber, tomato, lettuce, dried basil and oil)

b. beef stew with buckwheat groats

c. letcho with chicken and white rice

d. sour cucumber soup

e. tomato soup with pasta

f. pancakes with cottage cheese and jam

g. dumplings with cheese and potatoes

h. croquette with cabbage and mushrooms

i. cod fried in butter with garlic and dill

38. please mark two of the following products that you find most appetizing.

a. chocolate ice cream

b. vanilla ice cream

c. dark chocolate

d. vanilla pudding/cream pudding

e. yeast cake with crumble topping

f. sweet roll with berries

g. marquise cookies

39. please mark two of the following products that you find least appetizing.
    - a. chocolate ice cream
    - b. vanilla ice cream
    - c. dark chocolate
    - d. vanilla pudding/cream pudding
    - e. yeast cake with crumble topping
    - f. sweet roll with berries
    - g. marquise cookies

40 Please mark two of the following fruits that you find most appetizing.
    - a. strawberries
    - b. watermelon
    - c. banana
    - d. pineapple
    - e. peach
    - f. lemon
    - g. apple
    - h. pear
    - i. grape
    - j. orange

41. Please mark two of the following fruits that you find least appetizing.
    - k. strawberries
    - l. watermelon
    - m. banana
    - n. pineapple
    - o. peach
    - p. lemon
    - q. apple
    - r. pear
    - s. grape
    - t. orange

42. Please mark two of the following fragrances that you find most appetizing.
    - a. coffee
    - b. black tea
    - c. cinnamon

d. vanilla

e. rosemary

f. kefir

g. grilled sausage

h. roast beef

i. naturally smoked bacon

j. maple syrup

k. cream cheese

43. Please mark two of the following smells that you find least appetizing.

a. coffee

b. black tea

c. cinnamon

d. vanilla

e. rosemary

f. kefir

g. grilled sausage

h. roast beef

i. naturally smoked bacon

j. maple syrup

k. cream cheese

44. Please mark two of the following foods that you find the most filling.

a. vegetable salad (consisting of cucumber, tomato, lettuce and oil)

b. beef stew with buckwheat groats

c. letcho with chicken and white rice

d. sour cucumber soup

e. tomato soup with noodles

f. pancakes with cottage cheese and jam

g. dumplings with cheese and potatoes

h. croquette with cabbage and mushrooms

i. cod fried in butter with garlic and dill

45. Please mark two of the following foods that you find least filling.

a. vegetable salad (consisting of cucumber, tomato, lamb's lettuce and oil)

b. beef stew with buckwheat groats

c. letcho with chicken and white rice

d. sour cucumber soup

e. tomato soup with noodles

f. pancakes with cottage cheese and jam

g. dumplings with cheese and potatoes

h. croquette with cabbage and mushrooms

i. cod fried in butter with garlic and dill

46. Please mark two of the following foods that you find most filling.

a. chocolate ice cream

b. vanilla ice cream

c. dark chocolate

d. vanilla pudding/cream pudding

e. yeast crumble cake

f. sweet roll with berries

g. marquise cookies

47. Please mark two of the following products that you find the least filling.

a. chocolate ice cream

b. vanilla ice cream

c. dark chocolate

d. vanilla pudding/cream pudding

e. yeast cake with crumble topping

f. sweet roll with berries

g. marquise cookies

48. Please mark two of the following fruits that you find the most filling.

a. strawberries

b. watermelon

c. banana

d. pineapple

e. peach

f. lemon

g. apple

h. pear

i. grape

j. orange

49. Please mark two of the following fruits that you find least satiating.

a. strawberries
b. watermelon
c. banana
d. pineapple
e. peach
f. lemon
g. apple
h. pear
i. grape
j. orange

50. Please mark two of the following fragrances that you find most filling.

a. coffee
b. black tea
c. cinnamon
d. vanilla
e. rosemary
f. kefir
g. grilled sausage
h. roast beef
i. naturally smoked bacon
j. maple syrup
k. cream cheese

51. please mark two of the following smells that you find the least filling.

a. coffee
b. black tea
c. cinnamon
d. vanilla
e. rosemary
f. kefir
g. grilled sausage
h. roast beef
i. naturally smoked bacon
j. maple syrup
k. cream cheese

52. Is there any aroma after which, while eating a meal, you would feel faster You would feel full? If yes, what is it?

  a. yes (what?) ....

  b. no

53. Is there any smell after which you would feel hungry? If yes then what kind?

  a. yes (what?) ....

  b. no
