# Supplementary material for: Natural Appetite Control: Consumer Perception of Food-Based Appetite Regulating Aromas
Source: Nutrients. 2023 Jun 30;15(13):2996. doi: 10.3390/nu15132996 (PMC10347076; doi:10.3390/nu15132996)
Supplement: Supplementary file 1 [file nutrients-15-02996-s001.zip › Supplementary materials File 4 - Sensory assessment questionnaire.pdf]

UNIWERSYTET PRZYRODNICZY WE WROCŁAWIU

LIDER PROJEKTU
Uniwersytet Przyrodniczy we Wrocławiu
Ul. C.K. Norwida 25
50-375 Wrocław

Sample code: ……………………. **Name & age:** ……………………………………….

Lider Prototypes - sensory panel

Dear Participants,
in the first place please accept our thanks for will of participating in this panel. The sensory evaluation of prototypes of appetite regulators is carried out within the framework of the project "New generation appetite active agents- useful for long-term care and weight control" supported by the National Centre for Research and Development, LIDER XI programme no. LIDER/48/0191/L-11/19/NCBR/2020.
You will receive a number of coded samples, which should be evaluated according to following questionnaire. Please give honest answers and do not consult them with other panel participants.
If you will have any doubts or questions, please do not hesitate to ask the leader of the panel for more information.
Please note, that the data obtained during sensory panel will be used, by project research group members, for publication and patenting purposes.
On behalf of research group,
Jacek Łyczko, project manager

1. **First association upon smelling the sample:**

……
……………………………………………………………………………………………

2. **Rate the fragrance intensity:**

- ☐ Undetectable
- ☐ Subtle
- ☐ Perceptible
- ☐ Intense
- ☐ Very intense

3. **With what do you associate the scent (a room in your home, a public place, a specific object, some specific situation or time of day etc.)?**

……
……
……

4. **Rate how pleasant you find the scent:**

- ☐ Repulsive
- ☐ Irritating
- ☐ Neutral
- ☐ Pleasant
- ☐ Very pleasant

Narodowe Centrum Badań i Rozwoju

„Nowej generacji środki regulujące apetyt – użyteczne w opiece długoterminowej oraz kontroli masy ciała" Akronim RANG.e
Projekt finansowany przez Narodowe Centrum Badań i Rozwoju
Numer umowy: LIDER XI

LIDER

5. What aroma notes do you identify with this sample (please specify minimum 1, maximum 3)?

……

6. How does the fragrance affect you (annoying, relaxing, stimulating, etc.)?

……..

7. What is your overall opinion about the strength of this fragrance?

- ☐ Adequate
- ☐ Too weak
- ☐ Too strong

8. In your opinion, has the sample the potential to affect appetite?

- ☐ Yes, to increase the appetite
- ☐ Yes, to reduce appetite
- ☐ No

9. If you observed an increase in appetite, evaluate the intensity of the effect:

- ☐ Undetectable
- ☐ Subtle
- ☐ Perceptible
- ☐ Intense
- ☐ Very intense

10. If you have observed a reduction in appetite, evaluate the intensity of the effect:

- ☐ Undetectable
- ☐ Subtle
- ☐ Perceptible
- ☐ Intense
- ☐ Very intense

11. If you have observed an increase in appetite, does it increase appetite for specific foods? If yes, which ones (sweets, diary, cereals, meat, vegetables, fruits etc.)?

……..

12. If you have observed a reduction in appetite, does it reduce appetite for specific foods? If yes, which ones (sweets, diary, cereals, meat, vegetables, fruits etc.)?

………………………………………………………………………………………………….........

Narodowe Centrum Badań i Rozwoju

„Nowej generacji środki regulujące apetyt – użyteczne w opiece długoterminowej oraz kontroli masy ciała" Akronim RANG.e

Projekt finansowany przez Narodowe Centrum Badań i Rozwoju
Numer umowy: LIDER XI

LIDER
