# Supplementary material for: Natural Appetite Control: Consumer Perception of Food-Based Appetite Regulating Aromas
Source: Nutrients. 2023 Jun 30;15(13):2996. doi: 10.3390/nu15132996 (PMC10347076; doi:10.3390/nu15132996)
Supplement: Supplementary file 1 [file nutrients-15-02996-s001.zip › Supplementary materials File 5 - statistical analysis.pdf]

Translated from Polish to English - www.onlinedoctranslator.com

Table 1. Characteristics of the study group including the assessment of their own figure and BMI [kg/m2] (n = 530)

BMI category [kg/m2]	Silhouette number 1		Silhouette number 2		Silhouette number 3		Silhouette number 4		Silhouette number 5		Total		p
	n	%	n	%	n	%	n	%	n	%	n	%	
Underweight	2	40.00	3	60.00	-	-	-	-	-	-	5	0.94	0.0000
Correct body weight	8	6.84	**85**	**72.65**	**23**	**19.66**	1	0.85	-	-	117	22.08	
Overweight	-	-	**23**	**11.11**	**141**	**68.12**	**42**	**20.29**	1	0.48	207	39.06	
I degree obesity	-	-	3	2.29	**44**	**33.59**	**78**	**59.54**	6	4.58	131	24.72	
Grade II obesity	-	-	-	-	6	13.64	**28**	**63.64**	10	22.73	44	8.30	
Grade III obesity	-	-	-	-	1	3.85	17	65.38	8	30.77	26	4.90	
Together	10	1.89	114	21.51	215	40.57	166	31.32	25	4.72	530	100	

p - probability in the statistical chi2 test (significance level α < 0.05), statistically significant differences in bold

"Next Generation Appetite Control Agents - Useful for Long-Term Care and Weight Management" Acronym RANG.e

Narodowe Centrum Badań i Rozwoju

Project financed by the National Center for Research and Development Contract number: LIDER/48/0191/L-11/19/NCBR/2020

LIDER

The study used the Simplified Nutritional Appetite Questionnaire (sNaQ), which is used to assess appetite and the risk of weight loss, which is an extremely important element of the overall assessment of the nutritional status of patients.

Abnormal sNaQ result (score ≤ 14 points, which means the risk of further weight loss within 6 months) was found in 21.5% of the study group, including 73 women and 41 men. Taking into account the age of the subjects, the risk of weight loss was observed in 39 people aged 18-59 and in 75 people aged ≥ 60 years.

Table 2. Characteristics of the study group, taking into account the Simplified Appetite Questionnaire (sNaQ) and gender (n = 530)

	sNaQ classification					
Sex	1*		2*		Together	
	n	%	n	%	n	%
Women	73	22.26	255	77.74	328	61.89
Men	41	20.30	161	79.70	202	38.11
Total	114	21.51	416	78.49	530	100

* sum of points obtained: 1 - ≤ 14 points; 2 - (14-20> points

Table 3. Characteristics of the study group including the Simplified Appetite Questionnaire (sNaQ) and age (n = 530)

Age	sNaQ classification					
	1*		2*		Together	
	n	%	n	%	n	%
18-59 years old	45	17.65	210	82.35	255	48.11
≥ 60 years	69	25.09	206	74.91	275	51.89
Total	114	21.51	416	78.49	530	100

* sum of points obtained: 1 - ≤ 14 points; 2 - (14-20> points

Table 4. Characteristics of the study group including the Simplified Appetite Assessment Questionnaire (sNaQ) and BMI [kg/m2] (n = 530)

BMI classification [kg/m2]	sNaQ classification					
	1*		2*		Together	
	n	%	n	%	n	%
Malnutrition	1	20.00	4	80.00	5	0.94
Correct BMI	**38**	**32.48**	**79**	**67.52**	117	22.08

Overweight	**43**	**20.77**	**164**	**79.23**	207	39.06
I degree obesity	**19**	**14.50**	**112**	**85.50**	131	24.72
Grade II obesity	10	22.73	34	77.27	44	8.30
Grade III obesity	3	11.54	23	88.46	26	4.91
Total	114	21.51	416	78.49	530	100

* sum of points obtained: 1 - ≤ 14 points; 2 - (14-20> points

A very high, positive correlation (0.76) was found between the weight-height index (BMI [kg/m2]) and the perception of one's own figure among the respondents, therefore BMI and gender were assumed as the grouping (independent) variable. There was also a positive but weak correlation (0.13) between the BMI value and the sum of the points obtained in the sNaQ questionnaire.

There was also a weak, positive correlation (0.11) between BMI [kg/m2] and hunger suppression after drinking tea with sugar.

Narodowe Centrum Badań i Rozwoju

"Next Generation Appetite Control Agents - Useful for Long-Term Care and Weight Management" Acronym RANG.e
Project financed by the National Center for Research and Development Contract number: LIDER/48/0191/L-11/19/NCBR/2020

LIDER

Question 5.
Do you feel full (hunger suppressed) after drinking a glass of bitter black tea?

Table 5. The feeling of satiety after drinking bitter black tea and the weight-height index of the subjects (n=530)

	Yes		*Moderation*		*NO*		*I do not consume this product*		*Together*		*p*
BMI category [kg/m2]	*n*	*%*	*n*	*%*	*n*	*%*	*n*	*%*	*n*	*%*	
Underweight	2	40.00	-	-	3	60.00	-	-	5	0.94	
Correct body weight	6	5.13	**18**	**15.38**	**72**	**61.54**	**21**	**17.95**	117	22.08	
Overweight	**17**	**8.21**	**44**	**21.26**	**81**	**39.13**	**65**	**31.40**	207	39.06	**0.00543**
I degree obesity	**15**	**11.45**	**31**	**23.66**	**55**	**41.98**	**thirty**	**22.90**	131	24.72	
Grade II obesity	6	13.64	9	20.45	**15**	**34.09**	**14**	**31.82**	44	8.30	
Grade III obesity	1	3.85	6	23.08	10	38.46	9	34.62	26	4.90	
Total	47	8.87	108	20.38	236	44.53	139	26.23	530	100.00	

Narodowe Centrum Badań i Rozwoju

"Next Generation Appetite Control Agents - Useful for Long-Term Care and Weight Management" Acronym RANG.e
Project financed by the National Center for Research and Development Contract number: LIDER/48/0191/L-11/19/NCBR/2020

LIDER

p - probability in the statistical chi2 test (significance level α < 0.05), statistically significant differences in bold

Question 6.
After drinking a glass of black tea sweetened with 1 teaspoon of white sugar, do you feel full (hunger suppressed)?

Table 6. The feeling of satiety after drinking sweetened black tea and the weight-height index of the subjects (n=530)

	Yes		*Moderation*		*NO*		*I do not consume this product*		*Together*		*p*
BMI category [kg/m2]	*n*	*%*	*n*	*%*	*n*	*%*	*n*	*%*	*n*	*%*	
Underweight	-	-	1	20.00	2	40.00	2	40.00	5	0.94	**0.00030**
Correct body weight	**15**	**12.82**	**11**	**9.40**	**62**	**52.99**	**29**	**24.79**	117	22.08	
Overweight	**21**	**10.14**	**38**	**18.36**	**54**	**26.09**	**94**	**45.41**	207	39.06	
I degree obesity	**14**	**10.69**	**23**	**17.56**	**39**	**29.77**	**55**	**41.98**	131	24.72	
Grade II obesity	1	2.27	6	13.64	**11**	**25.00**	**26**	**59.09**	44	8.30	
Grade III obesity	1	3.85	3	11.54	**12**	**46.15**	10	38.46	26	4.90	

"Next Generation Appetite Control Agents - Useful for Long-Term Care and Weight Management" Acronym RANG.e
Narodowe Centrum Badań i Rozwoju
Project financed by the National Center for Research and Development Contract number: LIDER/48/0191/L-11/19/NCBR/2020
LIDER

Total	52	9.81	82	15.47	180	33.96	216	40.75	530	100.00	

p - probability in the statistical chi2 test (significance level α < 0.05), statistically significant differences in bold

Question 7.
After drinking a glass of black tea with lemon juice, sweetened with 1 teaspoon of white sugar, do you feel full (hunger suppressed)?

Table 7. The feeling of satiety after drinking sweetened black tea with the addition of lemon, and the weight-height index of the subjects (n=530)

	Yes		*Moderation*		*NO*		*I do not consume this product*		*Together*		*p*
BMI category [kg/m2]	*n*	*%*	*n*	*%*	*n*	*%*	*n*	*%*	*n*	*%*	
Underweight	1	20.00	1	20.00	2	40.00	1	20.00	5	0.94	**0.00084**
Correct body weight	10	8.55	**13**	**11.11**	**65**	**55.56**	**29**	**24.79**	117	22.08	
Overweight	**23**	**11.11**	**44**	**21.26**	**60**	**28.99**	**80**	**38.65**	207	39.06	
I degree obesity	**14**	**10.69**	**25**	**19.08**	**35**	**26.72**	**57**	**43.51**	131	24.72	
Grade II obesity	3	6.82	8	18.18	**12**	**27.27**	**21**	**47.73**	44	8.30	

Grade III obesity	1	3.85	8	30.77	6	23.08	**11**	**42.31**	26	4.90	
Total	52	9.81	99	18.68	180	33.96	199	37.55	530	100.00	

p - probability in the statistical chi2 test (significance level $\alpha < 0.05$), statistically significant differences in bold

Question 8.
Do you feel hungry after drinking a glass of black tea sweetened with 1 teaspoon of white sugar (or other sweetener)?

Table 8. The feeling of hunger after drinking sweetened black tea and the weight-height index of the subjects (n=530)

	Yes		*Moderation*		*NO*		*I do not consume this product*		*Together*		*p*
BMI category [kg/m2]	*n*	*%*	*n*	*%*	*n*	*%*	*n*	*%*	*n*	*%*	**0.00007**
Underweight	-	-	1	20.00	3	60.00	1	20.00	5	0.94	
Correct body weight	5	4.27	8	6.84	**76**	**64.96**	**28**	**23.93**	117	22.08	
Overweight	8	3.86	**33**	**15.94**	**77**	**37.20**	**89**	**43.00**	207	39.06	
I degree obesity	9	6.87	**25**	**19.08**	**44**	**33.59**	**53**	**40.46**	131	24.72	

Grade II obesity	3	6.82	5	11.36	**13**	**29.55**	**23**	**52.27**	44	8.30	
Grade III obesity	2	7.69	7	26.92	6	23.08	**11**	**42.31**	26	4.90	
Total	27	5.09	79	14.91	219	41.32	205	38.68	530	100.00	

p - probability in the statistical chi2 test (significance level α < 0.05), statistically significant differences in bold

Question 9.
Do you feel full (hunger suppressed) after drinking a glass of green tea?

Table 9. The feeling of satiety after drinking green tea and the weight-height index of the subjects (n=530)

	Yes		*Moderation*		*NO*		*I do not consume this product*		*Together*		*p*
BMI category [kg/m2]	*n*	*%*	*n*	*%*	*n*	*%*	*n*	*%*	*n*	*%*	0.38462
Underweight	2	40.00	-	-	1	20.00	2	40.00	5	0.94	
Correct body weight	7	5.98	**15**	**12.82**	**59**	**50.43**	**36**	**30.77**	117	22.08	
Overweight	**24**	**11.59**	**33**	**15.94**	**84**	**40.58**	**66**	**31.88**	207	39.06	

"Next Generation Appetite Control Agents - Useful for Long-Term Care and Weight Management" Acronym RANG.e

Narodowe Centrum Badań i Rozwoju

Project financed by the National Center for Research and Development Contract number: LIDER/48/0191/L-11/19/NCBR/2020

LIDER

I degree obesity	**13**	**9.92**	**22**	**16.79**	**46**	**35.11**	**50**	**38.17**	131	24.72	
Grade II obesity	2	4.55	8	18.18	**18**	**40.91**	**16**	**36.36**	44	8.30	
Grade III obesity	2	7.69	4	15.38	**11**	**42.31**	9	34.62	26	4.90	
Total	50	9.43	82	15.47	219	41.32	179	33.77	530	100.00	

p - probability in the statistical chi2 test (significance level α < 0.05), statistically significant differences in bold

Question 10.
After drinking a glass of yerba mate, do you feel full (hunger suppressed)?

Table 10. The feeling of satiety after drinking yerba mate and the weight-height index of the subjects (n=530)

	Yes		*Moderation*		*NO*		*I do not consume this product*		*Together*		*p*
BMI category [kg/m2]	*n*	*%*	*n*	*%*	*n*	*%*	*n*	*%*	*n*	*%*	0.65516
Underweight	-	-	1	20.00	-	-	4	80.00	5	0.94	
Correct body weight	3	2.56	8	6.84	**15**	**12.82**	**91**	**77.78**	117	22.08	

"Next Generation Appetite Control Agents - Useful for Long-Term Care and Weight Management" Acronym RANG.e
Narodowe Centrum Badań i Rozwoju
Project financed by the National Center for Research and Development Contract number: LIDER/48/0191/L-11/19/NCBR/2020
LIDER

Overweight	7	3.38	**17**	**8.21**	**47**	**22.71**	**136**	**65.70**	207	39.06	
I degree obesity	3	2.29	9	6.78	**29**	**22.14**	**90**	**68.70**	131	24.72	
Grade II obesity	2	4.55	2	4.55	6	13.64	**34**	**77.27**	44	8.30	
Grade III obesity	-	-	2	7.69	7	26.92	**17**	**65.38**	26	4.90	
Total	15	2.83	39	7.36	104	19.62	372	70.19	530	100.00	

p - probability in the statistical chi2 test (significance level α < 0.05), statistically significant differences in bold

Question 11.
Do you feel hungry after drinking a cup of coffee (without added milk and/or sugar)?

Table 11. The feeling of hunger after drinking an espresso and the weight-height index of the subjects (n=530)

	Yes		*Moderation*		*NO*		*I do not consume this product*		*Together*		*p*
BMI category [kg/m2]	*n*	*%*	*n*	*%*	*n*	*%*	*n*	*%*	*n*	*%*	0.25249
Underweight	-	-	1	20.00	2	40.00	2	40.00	5	0.94	

Correct body weight	6	5.13	**15**	**12.82**	**79**	**67.52**	**17**	**14.53**	117	22.08	
Overweight	**21**	**10.14**	**36**	**17.39**	**108**	**52.17**	**42**	**20.29**	207	39.06	
I degree obesity	**11**	**8.40**	**24**	**18.32**	**69**	**52.67**	**27**	**20.61**	131	24.72	
Grade II obesity	2	4.55	5	11.36	**24**	**54.55**	**13**	**29.55**	44	8.30	
Grade III obesity	4	15.38	2	7.69	**17**	**65.38**	3	11.54	26	4.90	
Total	44	8.30	83	15.66	299	56.42	104	19.62	530	100.00	

p - probability in the statistical chi2 test (significance level α < 0.05), statistically significant differences in bold

Question 12.
Do you feel full after eating a dish containing raw chili peppers?

Table 12. Feeling of satiety after eating a dish containing raw chili pepper, and the weight-height index of the subjects (n=530)

	Yes		*Moderation*		*NO*		*I do not consume this product*		*Together*		*p*
BMI category [kg/m2]	*n*	*%*	*n*	*%*	*n*	*%*	*n*	*%*	*n*	*%*	0.17722

Underweight	1	20.00	-	-	1	20.00	3	60.00	5	0.94	
Correct body weight	**11**	**9.40**	**25**	**21.37**	**22**	**18.80**	**59**	**50.43**	117	22.08	
Overweight	10	4.83	**37**	**17.87**	**60**	**28.99**	**100**	**43.31**	207	39.06	
I degree obesity	9	6.87	**26**	**19.85**	**31**	**23.66**	**65**	**49.62**	131	24.72	
Grade II obesity	4	9.09	3	6.82	9	20.45	**28**	**63.64**	44	8.30	
Grade III obesity	2	7.69	7	26.92	10	38.46	7	26.92	26	4.90	
Total	37	6.98	98	18.49	133	25.09	262	49.43	530	100.00	

p - probability in the statistical chi2 test (significance level α < 0.05), statistically significant differences in bold

Question 13.
Do you feel full after eating a product or dish containing chilli powder?

Table 13. Feeling of satiety aftereating a product/dish containing powdered chili pepper, and the weight-height index of the subjects (n=530)

	Yes	*Moderation*	*NO*	*I do not consume this product*	*Together*	*p*

BMI category [kg/m2]	*n*	*%*	*n*	*%*	*n*	*%*	*n*	*%*	*n*	*%*	
Underweight	1	20.00	-	-	2	40.00	2	40.00	5	0.94	
Correct body weight	**14**	**11.97**	**16**	**13.68**	**32**	**27.35**	**55**	**47.01**	117	22.08	
Overweight	**15**	**7.25**	**34**	**16.43**	**73**	**35.27**	**85**	**41.06**	207	39.06	0.27421
I degree obesity	**12**	**9.16**	**24**	**18.32**	**37**	**28.24**	**58**	**44.27**	131	24.72	
Grade II obesity	6	13.64	6	13.64	8	18.18	**24**	**54.55**	44	8.30	
Grade III obesity	2	7.69	3	11.54	**14**	**53.85**	7	26.92	26	4.90	
Total	50	9.43	83	15.66	166	31.32	231	43.58	530	100.00	

p - probability in the statistical chi2 test (significance level α < 0.05), statistically significant differences in bold

Question 14.

Do products containing vanilla flavor increase your appetite? If so, which of the following products cause this effect?

Statistically significant differences in appetite sensations were observed after exposure to vanilla-flavored products. In the case of respondents with a normal body weight, 41 people had no effect on appetite and 76 did. Among the 237 overweight and obese people, the smell did not affect food consumption, and 162 respondents declared such an impact. Nearly 1/3 of the respondents (27.6%;

146 people) would eat vanilla pudding after exposure to the smell of vanilla, and 22% of the respondents (117 people) would eat vanilla ice cream.

Table 14. Feelings afterproduct containing vanilla aroma, and the weight-height index of the tested persons (n=530)

		Underweight	*Correct body weight*	*Overweight*	*I degree obesity*	*Grade II obesity*	*Grade III obesity*	*Total*	*p*
No, it doesn't increase the appetite	*n*	2	**41**	**122**	**70**	**28**	**17**	**280**	0.000 52
	%	40.00	**35.04**	**58.94**	**53.44**	**63.64**	**65.38**	**52.83**	
Vanilla flavored tea	*n*	-	6	6	5	1	-	18	0.768 07
	%	-	5.13	2.90	3.82	2.27	-	3.40	
Vanilla pudding	*n*	3	**51**	**44**	**36**	6	6	146	0.000 08
	%	60.00	**43.59**	**21.26**	**27.48**	13.64	23.08	27.55	
Cake with the addition of vanilla oil	*n*	2	**14**	**34**	**31**	8	5	94	0.166 81
	%	40.00	**11.97**	**16.43**	**23.66**	18.18	19.23	17.74	
Vanilla ice cream	*n*	2	**thirty**	**38**	**29**	**11**	7	117	0.544 22
	%	40.00	**25.64**	**18.36**	**22.14**	**25.00**	26.92	22.08	

p - probability in the statistical chi2 test (significance level α < 0.05), statistically significant differences in bold

Question 15.
What are your feelings after eating a product/dish with nutmeg?

Table 15. Feelings aftereating a product/dish containing nutmeg and the weight-height index of the subjects (n=530)

	Definitely increases apatite		*Increases apatite*		*It does not affect the appetite*		*Lowers apatite*		*Definitely lowers apatite*		*Together*		*p*
BMI category [kg/m2]	*n*	*%*	*n*	*%*	*n*	*%*	*n*	*%*	*n*	*%*	*n*	*%*	
Underweight	-	-	1	20.00	4	80.00	-	-	-	-	5	0.94	0.76762
Correct body weight	1	0.85	8	6.84	**103**	**88.03**	4	3.42	1	0.85	117	22.08	
Overweight	3	1.45	**17**	**8.21**	**169**	**81.64**	**13**	**6.28**	5	2.45	207	39.06	
I degree obesity	4	3.05	**13**	**9.92**	**104**	**79.39**	8	6.11	2	1.53	131	24.72	
Grade II obesity	2	4.55	4	9.09	**34**	**77.27**	4	9.09	-	-	44	8.30	
Grade III obesity	1	3.85	-	-	**24**	**92.31**	-	-	1	3.85	26	4.90	
Total	11	2.08	43	8.11	438	82.64	29	5.47	9	1.70	530	100.00	

p - probability in the statistical chi2 test (significance level α < 0.05), statistically significant differences in bold

Question 16.
What are your feelings after eating a product with the scent of cloves or a dish with the addition of cloves?

Table 16. Feelings aftereating a product/dish containing cloves, and the weight-height index of the examined persons (n=530)

	Definitely increases apatite		*Increases apatite*		*It does not affect the appetite*		*Lowers apatite*		*Definitely lowers apatite*		*Together*		*p*
BMI category [kg/m2]	*n*	*%*	*n*	*%*	*n*	*%*	*n*	*%*	*n*	*%*	*n*	*%*	
Underweight	1	20.00	-	-	3	60.00	1	20.00	-	-	5	0.94	
Correct body weight	-	-	**14**	**11.97**	**83**	**70.94**	**14**	**11.97**	6	5.13	117	22.08	
Overweight	1	0.48	**25**	**12.08**	**156**	**75.36**	**22**	**10.63**	3	1.45	207	39.06	**0.00000**
I degree obesity	-	-	**15**	**11.45**	**95**	**72.52**	**13**	**9.92**	8	6.11	131	24.72	
Grade II obesity	-	-	9	20.45	**31**	**70.45**	3	6.82	1	2.27	44	8.30	
Grade III obesity	-	-	3	11.54	**19**	**73.08**	3	11.54	1	3.85	26	4.90	

Total	2	0.38	66	12.45	387	73.02	56	10.57	19	3.58	530	100.00	

p - probability in the statistical chi2 test (significance level α < 0.05), statistically significant differences in bold

Question 17.
What are your feelings after eating a cinnamon-scented product or a dish with cinnamon?

Table 17. Feelings aftereating a product/dish containing cinnamon and the weight-height index of the subjects (n=530)

	Definitely increases apatite		*Increases apatite*		*It does not affect the appetite*		*Lowers apatite*		*Definitely lowers apatite*		*Together*		*p*
BMI category [kg/m2]	*n*	*%*	*n*	*%*	*n*	*%*	*n*	*%*	*n*	*%*	*n*	*%*	
Underweight	1	20.00	1	20.00	3	60.00	-	-	-	-	5	0.94	0.08814
Correct body weight	3	2.56	**29**	**24.79**	**60**	**51.28**	**23**	**19.66**	2	1.71	117	22.08	
Overweight	8	3.86	**56**	**27.05**	**123**	**59.42**	**18**	**8.70**	2	0.97	207	39.06	
I degree obesity	2	1.53	**32**	**24.43**	**77**	**58.78**	**16**	**12.21**	4	3.05	131	24.72	
Grade II obesity	4	9.09	8	18.18	**25**	**56.82**	4	9.09	3	6.82	44	8.30	

Grade III obesity	2	7.69	4	15.38	**17**	**65.38**	2	7.69	1	3.85	26	4.90	
Total	20	3.77	130	24.53	305	57.55	63	11.89	12	2.26	530	100.00	

p - probability in the statistical chi2 test (significance level α < 0.05), statistically significant differences in bold

Question 18.
After exposure to the smell of bananas, do you feel like eating any of the following products?

Table 18. Feelings after exposure to the smell of bananas and the weight-height index of the subjects (n=530)

		Underweight	*Correct body weight*	*Overweight*	*I degree obesity*	*Grade II obesity*	*Grade III obesity*	*Total*	*p*
Bananas	*n*	2	**77**	**104**	**60**	**21**	**16**	280	**0.02692**
	%	40.00	**65.81**	**50.24**	**45.80**	**47.73**	**61.54**	52.83	
Milk chocolate	*n*	-	**25**	**thirty**	**15**	3	1	74	0.05191
	%	-	**21.37**	**14.49**	**11.45**	6.82	3.85	13.96	
Bitter chocolate	*n*	-	10	**13**	7	2	1	33	0.84187
	%	-	8.55	**6.28**	5.34	4.55	3.85	6.23	
Cakes and cookies	*n*	-	**16**	**16**	8	7	7	54	**0.00876**
	%	-	**13.68**	**7.73**	6.11	15.91	26.92	10.19	

vanilla yogurt	*n*	1	**11**	**15**	**11**	3	6	47	0.14341
	%	20.00	**9.40**	**7.25**	**8.40**	6.82	23.08	8.87	
Natural yogurt	*n*	2	7	**11**	6	5	-	31	**0.00983**
	%	40.00	5.98	**5.31**	4.58	11.36	-	5.85	
Jam	*n*	1	2	3	2	1	1	10	0.08227
	%	20.00	1.71	1.45	1.53	2.27	3.85	1.89	
Crackers	*n*	-	1	5	2	-	-	8	0.75679
	%	-	0.85	2.42	1.53	-	-	1.51	
Breadsticks	*n*	1	1	7	3	-	-	12	0.05242
	%	20.00	0.85	3.38	2.29	-	-	2.26	
Chips	*n*	-	2	9	1	1	2	15	0.24124
	%	-	1.71	4.35	0.76	2.27	7.69	2.83	
I don't feel like eating anything	*n*	1	**24**	**71**	**51**	**16**	6	169	**0.03244**
	%	20.00	**20.51**	**34.30**	**38.93**	**36.36**	23.08	31.89	

p - probability in the statistical chi2 test (significance level $\alpha < 0.05$), statistically significant differences in bold

Question 19.
After exposure to the smell of watermelon, do you feel like eating any of the following products?

Table 19. Feelings after exposure to the smell of watermelons and the weight-height index of the subjects (n=530)

		Underweight	*Correct body weight*	*Overweight*	*I degree obesity*	*Grade II obesity*	*Grade III obesity*	*Total*	*p*
Watermelon	*n*	2	**38**	**46**	**38**	**12**	7	143	0.43076
	%	40.00	**32.48**	**22.22**	**29.01**	**27.27**	26.92	26.98	
Bananas	*n*	-	2	9	5	2	5	23	**0.00641**
	%	-	1.71	4.35	3.82	4.55	19.23	4.34	
Milk chocolate	*n*	-	4	**13**	4	1	2	24	0.57579
	%	-	3.42	**6.28**	3.05	2.27	7.69	4.53	
Bitter chocolate	*n*	-	5	8	3	2	2	20	0.81401
	%	-	4.27	3.86	2.29	4.55	7.69	3, 77	
Cakes and cookies	*n*	-	1	**11**	9	2	1	24	0.31858
	%	-	0.85	**5.31**	6.87	4.55	3.85	4.53	
vanilla yogurt	*n*	-	3	10	1	-	1	15	0.25190
	%	-	2.56	4.83	0.76	-	3.85	2.83	

NCBR Narodowe Centrum Badań i Rozwoju

„Nowej generacji środki regulujące apetyt – użyteczne w opiece długoterminowej oraz kontroli masy ciała" Akronim RANG.e
Projekt finansowany przez Narodowe Centrum Badań i Rozwoju
Numer umowy: LIDER/48/0191/L-11/19/NCBR/2020

LIDER

Natural yogurt	*n*	1	3	**12**	3	-	4	23	**0.00724**
	%	20.00	2.56	**5,080**	2.29	-	15.38	4.34	
Jam	*n*	1	3	9	4	1	2	20	0.32165
	%	20.00	2.56	4.35	3.05	2.27	7.69	3.77	
Crackers	*n*	-	3	1	2	-	1	7	0.48350
	%	-	2.56	0.48	1.53	-	3.58	1.32	
Breadsticks	*n*	1	1	4	1	-	-	7	**0.00870**
	%	20.00	0.85	1.93	0.76	-	-	1.32	
Chips	*n*	1	2	2	2	1	2	9	**0.04189**
	%	20.00	1.71	0.97	1.53	2.27	3.85	1.70	
I don't feel like eating anything	*n*	1	**65**	**112**	**70**	**27**	9	284	0.19885
	%	20.00	**55.56**	**54.11**	**53.44**	**61.36**	34.62	53.58	

p - probability in the statistical chi2 test (significance level $\alpha < 0.05$), statistically significant differences in bold

Question 20.
After exposure to the smell of vanilla/chocolate pudding, do you feel like eating any of the following products?

Table 20. Feelings after exposure to the smell of vanilla/chocolate pudding and the weight-height index of the subjects (n=530)

		Underweight	*Correct body weight*	*Overweight*	*I degree obesity*	*Grade II obesity*	*Grade III obesity*	*Total*	*p*
Vanilla/chocolate pudding	*n*	3	**67**	**44**	**32**	**13**	4	163	**0.00000**
	%	60.00	**57.26**	**21.26**	**24.43**	**25.99**	15.38	30.75	
Bananas	*n*	-	5	**13**	6	2	4	thirty	0.31977
	%	-	4.27	**6.28**	4.58	4.55	15.38	5.66	
Milk chocolate	*n*	-	**thirty**	**35**	**22**	8	**13**	108	**0.00123**
	%	-	**25.64**	**16.91**	**16.79**	18, 18	**50.00**	20.38	
Bitter chocolate	*n*	-	7	**12**	6	2	1	28	0.97784
	%	-	5.98	**5.80**	4.58	4.55	3.85	5.28	
Cakes and cookies	*n*	1	**22**	**26**	**18**	7	7	81	0.37287
	%	20.00	**18.80**	**12.56**	**13.74**	15.91	26.92	15.28	
vanilla yogurt	*n*	3	**46**	**40**	**23**	5	7	124	**0.00003**
	%	60.00	**39.32**	**19.32**	**17.56**	11.36	26.92	23.40	
Natural yogurt	*n*	-	5	10	3	-	1	19	0.61541
	%	-	4.27	4.83	2.29	-	3.85	3.58	

Jam	*n*	-	2	8	5	1	1	17	0.89954
	%	-	1.71	3.86	3.82	2.27	3.85	3.21	
Crackers	*n*	-	2	1	1	-	-	4	0.82112
	%	-	1.71	0.48	0.76	-	-	0.75	
Breadsticks	*n*	-	2	4	4	-	4	14	**0.00225**
	%	-	1.71	1.93	3.05	-	15.38	2.64	
Chips	*n*	-	2	5	5	-	2	14	0.40998
	%	-	1.71	2.42	3.82	-	7.69	2.64	
I don't feel like eating anything	*n*	1	**27**	**81**	**54**	**23**	5	191	**0.00143**
	%	20.00	**23.08**	**39.13**	**41.22**	**52.27**	19.23	36.04	

p - probability in the statistical chi2 test (significance level α < 0.05), statistically significant differences in bold

Question 21.
After exposure to the smell of raw vegetables, do you feel like eating them? If so, what kind of vegetables are they?

Table 21. Feelings after exposure to the smell of raw vegetables and the weight-height index of the subjects (n=530)

	Underweight	*Correct body weight*	*Overweight*	*I degree obesity*	*Grade II obesity*	*Grade III obesity*	*Together*	*p*

Brassica vegetables	*n*	4	**19**	**41**	**28**	**11**	4	107	**0.020 14**
	%	80.00	**16.24**	**19.81**	**21.37**	**25.00**	15.38	20.19	
Onion vegetables	*n*	-	**22**	**46**	**28**	8	5	109	0.835 36
	%	-	**18.80**	**22.22**	**21.37**	18.18	19.23	20.57	
Leafy vegetables	*n*	1	7	**32**	**18**	6	4	68	0.244 90
	%	20.00	5.98	**15.46**	**13.74**	13.64	15.38	12.83	
Root vegetables	*n*	4	**12**	**33**	**24**	4	3	80	**0.000 73**
	%	80.00	**10.26**	**15.94**	**18.32**	9.09	11.54	15.09	
Nightshade vegetables	*n*	2	62	70	51	17	7	209	**0.019 36**
	%	40.00	52.99	33.82	38.93	38.64	26.92	39.43	
Green cucumber	*n*	2	**52**	**65**	**51**	**14**	**11**	195	0.250 37
	%	40.00	**44.44**	**31.40**	**38.93**	**31.82**	**42.31**	36.79	
Pumpkin	*n*	1	1	5	4	1	1	13	0.150 87
	%	20.00	0.85	2.42	3.05	2.27	3.85	2.45	
Turnip vegetables	*n*	1	5	**18**	**11**	5	5	45	0.163 51
	%	20.00	4.27	**8.70**	**8.40**	11.36	19.23	8.49	

Mushrooms	*n*	1	**20**	**39**	**25**	**12**	5	102	0.82103
	%	20.00	**17.09**	**18.84**	**19.08**	**27.27**	19.23	19.25	
No, I don't feel like it	*n*	-	**16**	**59**	**37**	**12**	8	132	**0.03005**
	%	-	**13.68**	**28.50**	**28.24**	**27.27**	30.77	24.91	

p - probability in the statistical chi2 test (significance level α < 0.05), statistically significant differences in bold

Question 22.
After exposure to the smell of raw fruit, do you feel like eating them? If so, what kind of fruit are they?

Table 22. Feelings after exposure to the smell of raw fruit and the weight-height index of the subjects (n=530)

		Underweight	*Correct body weight*	*Overweight*	*I degree obesity*	*Grade II obesity*	*Grade III obesity*	*Together*	*p*
Citrus fruits	*n*	2	**43**	**99**	**63**	**20**	**13**	**240**	0.45563
	%	40.00	**36.75**	**47.83**	**48.09**	**45.45**	**50.00**	**45.28**	
Exotic fruits	*n*	3	10	**38**	**23**	5	9	88	**0.00137**
	%	60.00	8.55	**18.36**	**17.56**	11.36	34.62	16.60	
Figs/dates	*n*	2	**12**	**21**	**12**	5	8	60	**0.01072**
	%	40.00	**10.26**	**10.14**	**9.16**	11.36	30.77	11.32	

Apples/pears	*n*	4	**58**	**96**	**47**	**19**	10	234	0.13499
	%	80.00	**49.57**	**46.38**	**35.88**	**43.18**	38.46	44.15	
Cherries cherries	*n*	3	**26**	**80**	**41**	**19**	9	178	**0.02481**
	%	60.00	**22.22**	**38.65**	**31.30**	**43.18**	34.62	33.58	
Peaches/apricots/nectarines	*n*	3	**31**	**72**	**45**	**15**	10	176	0.46107
	%	60.00	**26.50**	**34.78**	**34.35**	**34.09**	38.46	33.21	
Berries	*n*	5	**77**	**118**	**75**	**25**	**12**	312	0.15222
	%	100.00	**65.81**	**57.00**	**57.25**	**56.82**	**46.15**	58.87	
Bananas	*n*	3	**16**	**42**	**28**	**11**	6	106	0.11181
	%	60.00	**13.68**	**20.29**	**21.37**	**25.00**	23.08	20.00	
No, I don't feel like it	*n*	-	6	**27**	**17**	7	3	60	0.22054
	%	-	5.13	**13.04**	**12.98**	15.91	11.54	11.32	

p - probability in the statistical chi2 test (significance level α < 0.05), statistically significant differences in bold

Question 23.
After exposure to the smell of cooked vegetables, do you feel like eating them? If so, what kind of vegetables are they?

Table 23. Feelings after exposure to the smell of cooked vegetables and the weight-height index of the subjects (n=530)

		Underweight	*Correct body weight*	*Overweight*	*I degree obesity*	*Grade II obesity*	*Grade III obesity*	*Together*	*p*
Brassica vegetables	*n*	4	**45**	**78**	**48**	**15**	6	196	0.26529
	%	80.00	**38.46**	**37.68**	**36.64**	**34.09**	23.08	36, 98	
Onion vegetables	*n*	-	7	**32**	**13**	1	5	58	**0.02101**
	%	-	5.98	**15.46**	**9.92**	2.27	19.23	10.94	
Leafy vegetables	*n*	2	**11**	**20**	**18**	3	2	56	0.20554
	%	40.00	**9.40**	**9.66**	**13.74**	6.82	7.69	10.57	
Root vegetables	*n*	1	**44**	**48**	**28**	10	6	137	0.05160
	%	20.00	**37.61**	**23.19**	**21.37**	22.73	23.08	25.85	
Nightshade vegetables	*n*	2	**14**	**40**	**22**	7	3	88	0.36898
	%	40.00	**11.97**	**19.32**	**16.79**	15.91	11.54	16.60	
Potatoes	*n*	5	**55**	**55**	**32**	**16**	4	167	**0.00000**
	%	100.00	**47.01**	**26.57**	**24.43**	**36.36**	15.38	31.51	
Pumpkin	*n*	2	6	**13**	6	-	2	29	**0.01121**
	%	40.00	5.13	**6.28**	4.58	-	7.69	5.47	

Mushrooms	*n*	1	**42**	**45**	**31**	**12**	6	137	0.12824
	%	20.00	**35.90**	**21.74**	**23.66**	**27.27**	23.08	25.85	
No, I don't feel like it	*n*	-	**20**	**59**	**39**	**16**	**12**	146	**0.01058**
	%	-	**17.09**	**28.50**	**29.77**	**36.36**	**46.15**	27.55	

p - probability in the statistical chi2 test (significance level α < 0.05), statistically significant differences in bold

Question 24.
After exposure to the smell of fresh tomatoes, do you feel like eating tomato soup?

Table 24. Feelings after exposure to the smell of fresh tomatoes and the weight-height index of the subjects (n=530)

	Yes		*NO*		*Total*		*p*
BMI category [kg/m2]	*n*	*%*	*n*	*%*	*n*	*%*	
Underweight	3	60.00	2	40.00	5	0.94	0.13983
Correct body weight	**52**	**44.44**	**65**	**55.56**	117	22.08	
Overweight	**117**	**56.52**	**90**	**43.48**	207	39.06	
I degree obesity	**62**	**47.33**	**69**	**52.67**	131	24.72	

NCBR Narodowe Centrum Badań i Rozwoju

„Nowej generacji środki regulujące apetyt – użyteczne w opiece długoterminowej oraz kontroli masy ciała" Akronim RANG.e
Projekt finansowany przez Narodowe Centrum Badań i Rozwoju
Numer umowy: LIDER/48/0191/L-11/19/NCBR/2020

LIDER

Grade II obesity	**18**	**40.91**	**26**	**59.09**	44	8.30	
Grade III obesity	10	38.46	**16**	**61.54**	26	4.90	
Total	262	49.43	268	50.57	530	100	

p - probability in the statistical chi2 test (significance level α < 0.05), statistically significant differences in bold

Question 25.
After walking past a confectionery/bakery with the aroma of bread, do you feel like eating any of the following products?

Table 25. Feelings after exposure to smells floating next to the bakery/confectionery, and the weight-height index of the subjects (n=530)

		Underweight	*Correct body weight*	*Overweight*	*I degree obesity*	*Grade II obesity*	*Grade III obesity*	*Total*	*p*
Rye bread	*n*	3	**14**	**49**	**28**	10	1	105	**0.00638**
	%	60.00	**11.97**	**23.67**	**21.37**	22.73	3.85	19.81	
Graham bread	*n*	1	9	29	8	4	2	53	0.19615
	%	20.00	7.69	14.01	6.11	9.09	7.69	10.00	
Wheat bread	*n*	2	**27**	**39**	**29**	**17**	**13**	127	**0.00205**
	%	40.00	**23.08**	**18.84**	**22.14**	**38.64**	**50.00**	23.96	

Rye roll	*n*	1	**11**	**24**	**11**	6	4	57	0.77038
	%	20.00	**9.40**	**11.59**	**8.40**	13.64	15.38	10.57	
Graham	*n*	3	**21**	**35**	**15**	7	4	85	0.08527
	%	60.00	**17.95**	**16.91**	**11.45**	15.91	15.38	16.04	
Wheat roll	*n*	3	**54**	**75**	**45**	**14**	**13**	204	0.18249
	%	60.00	**46.15**	**36.23**	**34.35**	**31.82**	**50.00**	38.49	
Corn roll	*n*	-	7	7	9	-	3	26	0.19353
	%	-	5.98	3.38	6.87	-	11.54	4.91	
Sweet roll	*n*	4	**72**	**97**	**59**	**20**	**16**	268	**0.03735**
	%	80.00	**61.54**	**46.86**	**45.04**	**45.45**	**61.54**	50.57	
I don't feel like eating anything	*n*	-	3	**23**	**18**	2	2	48	**0.03238**
	%	-	2.56	**11.11**	**13.74**	4.55	7.69	9.06	
Other products	*n*	-	1	5	3	4	-	13	0.07117
	%	-	0.85	2.42	2.29	9.09	-	2.45	

p - probability in the statistical chi2 test (significance level $\alpha < 0.05$), statistically significant differences in bold

NCBR Narodowe Centrum Badań i Rozwoju

„Nowej generacji środki regulujące apetyt – użyteczne w opiece długoterminowej oraz kontroli masy ciała" Akronim RANG.e
Projekt finansowany przez Narodowe Centrum Badań i Rozwoju
Numer umowy: LIDER/48/0191/L-11/19/NCBR/2020

LIDER

Question 26.
After eating food with an intensely sweet taste (milk chocolate, chocolate bar, etc.) do you feel like eating any of the following products?

Table 26. Feelings after eating food with an intense sweet taste and the weight-height index of the subjects (n=530)

		Underweight	*Correct body weight*	*Overweight*	*I degree obesity*	*Grade II obesity*	*Grade III obesity*	*Total*	*p*
Pork chop	*n*	-	7	**25**	**11**	8	5	56	0.10474
	%	-	5.98	**12.08**	**8.40**	18.18	19.23	10.57	
Beef goulash	*n*	-	3	**10**	**12**	1	2	28	0.21144
	%	-	2.56	**4.83**	**9.16**	2.27	7.69	5.28	
Chicken fricassee	*n*	-	4	9	5	3	2	23	0.86183
	%	-	3.42	4.35	3.82	6.82	7.69	4.34	
Fried chicken	*n*	-	7	**15**	5	8	4	39	**0.02198**
	%	-	5.98	**7.25**	3.82	18.18	15.38	7.36	
Grilled chicken	*n*	-	6	**13**	7	5	3	34	0.57065
	%	-	5.13	**6.28**	5.34	11.36	11.54	6.42	
Groats	*n*	1	2	4	1	1	2	11	**0.02196**
	%	20.00	1.71	1.93	0.76	2.27	7.69	2.08	

NCBR Narodowe Centrum Badań i Rozwoju

„Nowej generacji środki regulujące apetyt – użyteczne w opiece długoterminowej oraz kontroli masy ciała" Akronim RANG.e
Projekt finansowany przez Narodowe Centrum Badań i Rozwoju
Numer umowy: LIDER/48/0191/L-11/19/NCBR/2020

LIDER

Rice	*n*	1	3	5	2	2	3	16	**0.02285**
	%	20.00	2.56	2.42	1.53	4.55	11.54	3.02	
Potatoes	*n*	1	5	7	4	2	2	21	0.43231
	%	20.00	4.27	3.38	3.05	4.55	7.69	3.96	
Natural yogurt	*n*	2	**25**	**14**	4	2	-	47	**0.00000**
	%	40.00	**21.37**	**6.76**	3.05	4.55	-	8.87	
Flavored yogurt	*n*	-	8	10	3	1	3	25	0.26768
	%	-	6.84	4.83	2.29	2.27	11.54	4.72	
Cheese	*n*	-	4	10	7	3	3	27	0.62370
	%	-	3.42	4.83	5.34	6.82	11.54	5.09	
Chocolate	*n*	1	**34**	**32**	**24**	8	6	105	0.10138
	%	20.00	**29.06**	**15.46**	**18.32**	18.18	23.08	19.81	
Cookies	*n*	2	**25**	**21**	**16**	3	4	71	**0.02130**
	%	40.00	**21.37**	**10.14**	**12.21**	6.82	15.38	13.40	
Crackers	*n*	-	7	**11**	4	3	3	28	0.55872
	%	-	5.98	**5.31**	3.05	6.82	11.54	5.28	

NCBR Narodowe Centrum Badań i Rozwoju

„Nowej generacji środki regulujące apetyt – użyteczne w opiece długoterminowej oraz kontroli masy ciała" Akronim RANG.e
Projekt finansowany przez Narodowe Centrum Badań i Rozwoju
Numer umowy: LIDER/48/0191/L-11/19/NCBR/2020

LIDER

Breadsticks	*n*	-	6	**17**	4	3	6	36	**0.00908**
	%	-	5.13	**8.21**	3.05	6.82	23.08	6.79	
Chips	*n*	1	10	**17**	6	5	6	45	0.05061
	%	20.00	8.55	**8.21**	4.58	11.36	23.08	8.49	
Fast food	*n*	-	4	**16**	**13**	4	8	45	**0.00066**
	%	-	3.42	**7.73**	**9.92**	9.09	30.77	8.49	
Other product/dish	*n*	-	4	4	7	2	-	17	0.50611
	%	-	3.42	1.93	5.34	4.55	-	3.21	
I don't feel like eating anything	*n*	2	**31**	**100**	**58**	**19**	8	218	**0.00550**
	%	40.00	**26.50**	**48.31**	**44.27**	**43.18**	30.77	41.13	

p - probability in the statistical chi2 test (significance level $\alpha < 0.05$), statistically significant differences in bold

Question 27.
After eating food with an intense umami flavor (with the addition of monosodium glutamate, e.g. Chinese soup, hot cup, stock cube) do you feel like eating any of the following products?

Table 27. Feelings after eating food with an intense umami taste, and the weight-height index of the subjects (n=530)

		Underweight	*Correct body weight*	*Overweight*	*I degree obesity*	*Grade II obesity*	*Grade III obesity*	*Total*	*p*

Pork chop	*n*	-	**17**	**20**	**20**	5	3	65	0.59281
	%	-	**14.53**	**9.66**	**15.27**	11.36	11.54	12.26	
Beef goulash	*n*	-	8	8	10	-	2	28	0.31824
	%	-	6.84	3.86	7.63	-	7.69	5.28	
Chicken fricassee	*n*	-	4	**13**	6	1	6	thirty	**0.00357**
	%	-	3.42	**6.28**	4.58	2.27	23.08	5.66	
Fried chicken	*n*	-	**11**	**16**	**17**	1	4	49	0.22278
	%	-	**9.40**	**7.73**	**12.98**	2.27	15.38	15.38	
Grilled chicken	*n*	-	5	**16**	**13**	4	4	42	0.37340
	%	-	4.27	**7.73**	**9.92**	9.09	15.38	7.92	
Groats	*n*	1	2	1	-	-	3	7	**0.00000**
	%	20.00	1.71	0.48	-	-	11.54	1.32	
Rice	*n*	-	3	5	2	1	1	12	0.97698
	%	-	2.56	2.42	1.53	2.27	3.85	2.26	
Potatoes	*n*	1	4	**11**	7	2	3	28	0.40870
	%	20.00	3.42	**5.31**	5.34	4.55	11.54	5.28	

NCBR Narodowe Centrum Badań i Rozwoju

„Nowej generacji środki regulujące apetyt – użyteczne w opiece długoterminowej oraz kontroli masy ciała" Akronim RANG.e
Projekt finansowany przez Narodowe Centrum Badań i Rozwoju
Numer umowy: LIDER/48/0191/L-11/19/NCBR/2020

LIDER

Natural yogurt	*n*	-	7	5	4	-	-	16	0.29628
	%	-	5.98	2.42	3.05	-	-	3.02	
Flavored yogurt	*n*	-	-	4	3	-	-	7	0.53393
	%	-	-	1.93	2.29	-	-	1.32	
Cheese	*n*	-	5	**12**	1	1	1	20	0.29160
	%	-	4.27	**5.80**	0.76	2.27	3.85	3.77	
Chocolate	*n*	-	4	**19**	**11**	1	2	37	0.29397
	%	-	3.42	**9.18**	**8.40**	2.27	7.69	6.98	
Cookies	*n*	-	2	**15**	5	3	2	27	0.29665
	%	-	1.71	**7.25**	3.82	6.82	7.69	5.09	
Crackers	*n*	-	3	9	3	1	3	19	0.26053
	%	-	2.59	4.35	2.29	2.27	11.54	3.58	
Breadsticks	*n*	-	3	5	3	1	4	16	**0.01328**
	%	-	2.56	2.42	2.29	2.27	15.38	3.02	
Chips	*n*	-	5	**19**	**11**	1	5	41	0.08352
	%	-	4.27	**9.18**	**8.40**	2.27	19.23	7.74	

Fast food	*n*	1	**17**	**22**	10	4	8	62	**0.02328**
	%	20.00	**14.53**	**10.63**	7.63	9.09	30.77	11.70	
I don't feel like eating anything	*n*	3	**59**	**117**	**71**	**28**	**11**	289	0.51686
	%	60.00	**50.43**	**53.52**	**54.20**	**63.64**	**42.31**	54.53	

p - probability in the statistical chi2 test (significance level α < 0.05), statistically significant differences in bold

Question 28.
After eating food with an intense salty taste, do you feel like eating any of the following products?

Table 28. Feelings after eating food with an intense salty taste and the weight-height index of the subjects (n=530)

		Underweight	*Correct body weight*	*Overweight*	*I degree obesity*	*Grade II obesity*	*Grade III obesity*	*Total*	*p*
Pork chop	*n*	-	**13**	**20**	**11**	3	3	50	0.90478
	%	-	**11.11**	**9.66**	**8.40**	6.82	11.54	9.43	
Beef goulash	*n*	-	8	**13**	7	1	-	29	0.64578
	%	-	6.84	**6.28**	5.34	2.27	-	5.47	
Chicken fricassee	*n*	-	2	8	3	1	1	15	0.87972
	%	-	1.71	3.86	2.29	2.27	3.85	2.83	
Fried chicken	*n*	-	**13**	**12**	9	2	1	37	0.45445

NCBR Narodowe Centrum Badań i Rozwoju

„Nowej generacji środki regulujące apetyt – użyteczne w opiece długoterminowej oraz kontroli masy ciała" Akronim RANG.e
Projekt finansowany przez Narodowe Centrum Badań i Rozwoju
Numer umowy: LIDER/48/0191/L-11/19/NCBR/2020

LIDER

	%	-	**11.11**	**5.80**	6.87	4.55	3.85	6.98	
Grilled chicken	*n*	-	7	**13**	8	3	-	31	0.83650
	%	-	5.98	**6.28**	6.11	6.82	-	5.85	
Groats	*n*	-	5	8	3	1	-	17	0.83650
	%	-	4.27	3.86	2.29	2.27	-	3.21	
Rice	*n*	1	3	5	3	1	1	14	0.29330
	%	20.00	2.56	2.42	2.29	2.27	3.85	2.64	
Potatoes	*n*	1	17	14	7	4	1	44	0.08344
	%	20.00	14.53	6.76	5.34	9.09	3.85	8.30	
Natural yogurt	*n*	1	**43**	8	**11**	1	1	65	**0.0000**
	%	20.00	**36.75**	3.86	**8.40**	2.27	3.85	12.26	
Flavored yogurt	*n*	-	9	**11**	5	1	1	27	0.67334
	%	-	7.69	**5.31**	3.82	2.27	3.85	5.09	
Cheese	*n*	-	5	10	6	2	-	23	0.90715
	%	-	4.27	4.83	4.58	4.55	-	4.34	
Chocolate	*n*	1	**20**	**33**	**21**	4	4	83	0.88655

NCBR Narodowe Centrum Badań i Rozwoju

„Nowej generacji środki regulujące apetyt – użyteczne w opiece długoterminowej oraz kontroli masy ciała" Akronim RANG.e
Projekt finansowany przez Narodowe Centrum Badań i Rozwoju
Numer umowy: LIDER/48/0191/L-11/19/NCBR/2020

LIDER

	%	20.00	**17.09**	**15.94**	**16.03**	9.09	15.38	15.66	
Cookies	*n*	1	8	**20**	**15**	4	5	53	0.45486
	%	20.00	6.84	**9.66**	**11.45**	9.09	19.23	10.00	
Crackers	*n*	-	2	**13**	5	4	7	31	**0.00006**
	%	-	1.71	**6.28**	3.82	9.09	16.92	5.85	
Breadsticks	*n*	-	3	**18**	9	3	6	39	**0.01341**
	%	-	1.56	**8.70**	6.87	6.82	23.08	7.36	
Chips	*n*	-	2	**25**	**12**	7	6	52	**0.00363**
	%	-	1.71	**12.08**	**9.16**	15.91	23.08	9.81	
Fast food	*n*	-	5	**13**	**12**	4	5	39	0.00363
	%	-	4.27	**6, 28**	**9.16**	9.09	19.23	7.36	
I don't feel like eating anything	*n*	2	**26**	**77**	**46**	**17**	6	174	0.07575
	%	40.00	**22.22**	**37.20**	**35.11**	**38.64**	23.08	32.83	

p - probability in the statistical chi2 test (significance level α < 0.05), statistically significant differences in bold

Question 29.
After eating food with added oil (chips, fried meat) do you feel like eating any of the following products?

Table 29. Feelings after eating food with added oil, and the weight-height index of the subjects (n=530)

		Underweight	*Correct body weight*	*Overweight*	*I degree obesity*	*Grade II obesity*	*Grade III obesity*	*Total*	*p*
Pork chop	*n*	-	**22**	**23**	**13**	3	4	65	0.17239
	%	-	**18.18**	**11.11**	**9.92**	6.82	15.38	12.26	
Beef goulash	*n*	-	1	4	8	2	2	17	0.11642
	%	-	0.85	1.93	6.11	4.55	7.69	3.21	
Chicken fricassee	*n*	-	5	9	6	2	1	23	0.99823
	%	-	4.27	4.35	4.58	4.55	385	4.34	
Fried chicken	*n*	-	**16**	**16**	9	3	2	46	0.39662
	%	-	**13.68**	**7, 73**	6.87	6.82	7.69	8.68	
Grilled chicken	*n*	-	5	**12**	9	3	3	32	0.76466
	%	-	4.27	**5.80**	6.87	6.82	11.54	6.04	
Groats	*n*	-	3	8	3	4	3	21	0.11997
	%	-	2.56	3.86	2.29	9.09	11.54	3.96	

Rice	*n*	-	3	6	10	3	2	24	0.25124
	%	-	2.56	2.90	7.63	6.82	7.69	4.53	
Potatoes	*n*	3	6	**16**	**13**	7	3	48	**0.00074**
	%	60.00	5.13	**7.73**	**9.92**	15.91	11.54	9.06	
Natural yogurt	*n*	1	7	7	7	1	1	24	0.45129
	%	20.00	5.98	3.38	5.34	2.27	3.85	4.53	
Flavored yogurt	*n*	-	5	**11**	3	1	1	21	0.77293
	%	-	4.27	**5.31**	2.29	2.27	3.85	3.96	
Cheese	*n*	-	2	**11**	3	3	2	21	0.34527
	%	-	1.71	**5.31**	2.29	6.82	7.69	3.96	
Chocolate	*n*	-	6	**22**	**14**	2	4	48	0.29806
	%	-	5.13	**10.63**	**10.69**	4.55	15.38	9.06	
Cookies	*n*	-	5	**20**	6	3	3	37	0.30786
	%	-	4.27	**9.66**	4.58	6.82	11.54	6.98	
Crackers	*n*	-	3	6	1	1	2	13	0.43377
	%	-	2.56	2.90	0.76	2.27	7.69	2.45	

Breadsticks	*n*	-	-	6	3	2	4	15	**0.00187**
	%	-	-	2.90	2.29	4.55	15.38	2.83	
Chips	*n*	-	5	**15**	10	3	3	36	0.74982
	%	-	4.27	**7.25**	7.63	6.82	11.54	6.79	
Fast food	*n*	-	**13**	**16**	7	3	3	42	0.57788
	%	-	**11.11**	**7.73**	5.34	6.82	11.54	7.92	
I don't feel like eating anything	*n*	2	**47**	**103**	**63**	**22**	5	242	0.05438
	%	40.00	**40.17**	**49.76**	**48.09**	**50.00**	19.23	45.66	

p - probability in the statistical chi2 test (significance level α < 0.05), statistically significant differences in bold

Question 30.
What are your feelings after exposure to the smell of green lettuce?

Table 30. Feelings after exposure to the smell of green lettuce and the weight-height index of the subjects (n=530)

		Underweight	*Correct body weight*	*Overweight*	*I degree obesity*	*Grade II obesity*	*Grade III obesity*	*Total*	*p*
Cravings for sweets are reduced	*n*	1	**48**	**25**	**16**	2	2	94	**0.00000**
	%	20.00	**41.03**	**12.08**	**12.21**	4.55	7.69	17.74	
The appetite for spicy	*n*	1	2	4	6	1	-	14	0.09763

products is reduced	%	20.00	1.71	1.93	4.58	2.27	-	2.64	
The appetite for flour products is reduced	*n*	2	9	**15**	**13**	1	-	40	**0.02838**
	%	40.00	7.69	**7.25**	**9.92**	2.27	-	7.55	
Appetite for poultry and/or meat is reduced	*n*	-	2	5	5	-	-	12	0.64640
	%	-	1.71	2.42	3.82	-	-	2.26	
The appetite for dairy products is reduced	*n*	1	**12**	8	8	1	1	31	0.12376
	%	20.00	**10.26**	3.86	6.11	2.27	3.85	5.85	
The appetite increasessweets	*n*	-	1	6	5	1	4	17	**0.01030**
	%	-	0.85	2.90	3.82	2.27	15.38	3.21	
Appetite increasesfor spicy products	*n*	-	2	-	-	-	-	2	0.21428
	%	-	1.71	-	-	-	-	0.38	
Appetite increasesfor flour products	*n*	1	1	6	3	-	4	15	**0.00029**
	%	20.00	0.85	2.90	2.29	-	15.38	2.83	
It increasescravings for poultry and/or meat	*n*	1	**15**	**23**	**18**	4	4	65	0.91538
	%	20.00	**12.82**	**11.11**	**13.74**	9.09	15.38	12.26	

NCBR Narodowe Centrum Badań i Rozwoju

„Nowej generacji środki regulujące apetyt – użyteczne w opiece długoterminowej oraz kontroli masy ciała" Akronim RANG.e
Projekt finansowany przez Narodowe Centrum Badań i Rozwoju
Numer umowy: LIDER/48/0191/L-11/19/NCBR/2020

LIDER

It increasescraving for dairy products	*n*	-	6	8	1	-	1	16	0.29224
	%	-	5.13	3.86	0.76	-	3.85	3.02	
N/A (no increase/decrease in appetite)	*n*	2	**53**	**134**	**80**	**36**	10	315	**0.00006**
	%	40.00	**45.30**	**64.73**	**61.07**	**81.82**	38.46	59.43	

p - probability in the statistical chi2 test (significance level α < 0.05), statistically significant differences in bold

Question 31.
How do you feel after exposure to the smell of dark chocolate?

Table 31. Feelings after exposure to dark chocolate and the weight-height index of the subjects (n=530)

		Underweight	*Correct body weight*	*Overweight*	*I degree obesity*	*Grade II obesity*	*Grade III obesity*	*Total*	*p*
Cravings for sweets are reduced	*n*	1	**21**	**58**	**18**	6	5	109	**0.02756**
	%	20.00	**17.95**	**28.02**	**13.74**	13.64	19.23	20.57	
The appetite for spicy products is reduced	*n*	-	8	**12**	6	-	-	26	0.39343
	%	-	6.84	**5.80**	4.58	-	-	4.91	
The appetite for flour products is reduced	*n*	-	4	**11**	4	-	1	20	0.62304
	%	-	3.42	**5.31**	3.05	-	3.85	3.77	
Appetite for poultry and/or	*n*	1	**11**	9	7	-	1	29	0.12061

meat is reduced	%	20.00	**9.40**	4.35	5.34	-	3.85	5.47	
The appetite for dairy products is reduced	n	-	4	**12**	4	-	-	20	0.35762
	%	-	3.42	**5.80**	3.05	-	-	3.77	
The appetite increasesswee ts	n	1	**69**	**46**	**36**	**14**	10	176	**0.00000**
	%	20.00	**58.97**	**22.22**	**27.48**	**31.82**	38.46	33.21	
Appetite increasesfor spicy products	n	-	1	-	1	-	2	4	**0.00221**
	%	-	0.85	-	0.76	-	7.69	0.75	
Appetite increasesfor flour products	n	1	4	7	5	-	2	19	0.23146
	%	20.00	3.42	3.38	3.82	-	7.69	3.58	
It increasescravi ngs for poultry and/or meat	n	-	2	5	3	1	3	14	0.12338
	%	-	1.71	2.42	2.29	2.27	11.54	2.64	
It increasescravi ng for dairy products	n	-	**34**	**14**	**18**	6	3	75	**0.00001**
	%	-	**29.06**	**6.76**	**13.74**	13.64	11.54	14.15	
N/A (no increase/decre ase in appetite)	n	2	**18**	**81**	**54**	**20**	5	180	**0.00003**
	%	40.00	**15.38**	**39.13**	**41.22**	**45.45**	19.23	33.96	

p - probability in the statistical chi2 test (significance level $\alpha < 0.05$), statistically significant differences in bold

Question 32. What are your feelings after exposure to the smell of yellow cheese?

Table 32. Feelings after exposure to the smell of yellow cheese and the weight-height index of the subjects (n=530)

		Underweight	*Correct body weight*	*Overweight*	*I degree obesity*	*Grade II obesity*	*Grade III obesity*	*Total*	*p*
Cravings for sweets are reduced	*n*	1	**15**	**20**	**11**	7	-	54	0.26425
	%	20.00	**12.82**	**9.66**	**8.40**	15.91	-	10.19	
The appetite for spicy products is reduced	*n*	-	2	3	4	-	-	9	0.72863
	%	-	1.71	1.45	3.05	-	-	1.70	
The appetite for flour products is reduced	*n*	-	3	8	4	1	1	17	0.97843
	%		2.56	3.86	3.05	2.27	3.85	3.21	
Appetite for poultry and/or meat is reduced	*n*	-	2	4	5	1	-	12	0.78984
	%	-	1.71	1.93	3.82	2.27	-	2.26	
The appetite for dairy products is reduced	*n*	-	4	**12**	5	-	1	22	0.58855
	%	-	3.42	**5.80**	3.82	-	3.85	4.15	
	n	-	4	**14**	3	-	5	26	**0.00298**

The appetite increasessweets	%	-	3.42	**6.76**	2.29	-	19.23	4.91	
Appetite increasesfor spicy products	*n*	-	10	**15**	**14**	5	5	49	0.39048
	%	-	8.55	**7.25**	**10.69**	11.36	19.23	9.25	
Appetite increasesfor flour products	*n*	1	6	**18**	**12**	2	5	44	0.18534
	%	20.00	5.13	**8.70**	**9.16**	4.55	19.23	8.30	
It increasescravings for poultry and/or meat	*n*	-	8	**22**	**13**	7	10	60	**0.00029**
	%	-	6.84	**10.63**	**9.92**	15.91	38.46	11.32	
It increasescraving for dairy products	*n*	3	**53**	**33**	**24**	10	4	127	**0.00000**
	%	60.00	**45.30**	**15.94**	**18.32**	22.73	15.38	23.96	
Cheese and products with its addition (pizza, casserole, toast)	*n*	-	-	2	3	3	-	8	**0.04138**
	%	-	-	0.97	2.29	6.82	-	1.51	
N/A (no increase/decrease in appetite)	*n*	1	**44**	**107**	**70**	**21**	8	251	**0.03047**
	%	20.00	**37.61**	**51.69**	**53.44**	**47.73**	30.77	47.36	

p - probability in the statistical chi2 test (significance level $\alpha < 0.05$), statistically significant differences in bold

Question 33.
What are your feelings after exposure to the smell of croquette with cabbage and mushrooms?

Table 33. Feelings after exposure to the smell of croquette with cabbage and mushrooms and the weight-height index of the subjects (n=530)

		Underweight	*Correct body weight*	*Overweight*	*I degree obesity*	*Grade II obesity*	*Grade III obesity*	*Total*	*p*
Cravings for sweets are reduced	*n*	3	**55**	**38**	**23**	10	1	130	**0.00000**
	%	60.00	**47.01**	**18.36**	**17.56**	22.73	3.85	24.53	
The appetite for spicy products is reduced	*n*	1	7	9	6	-	-	23	0.22751
	%	20.00	5.98	4.35	4.35	-	-	4.34	
The appetite for flour products is reduced	*n*	2	6	9	6	2	-	25	**0.00934**
	%	40.00	5.13	4.35	4.58	4.55	-	4.72	
Appetite for poultry and/or meat is reduced	*n*	1	4	8	6	-	2	21	0.27159
	%	20.00	3.42	3.86	4.58	-	7.69	3.96	
The appetite for dairy products is reduced	*n*	1	7	**19**	10	2	-	39	0.40458
	%	20.00	5.98	**9.18**	7.63	4.55	-	7.36	
The appetite increasessweets	*n*	-	2	6	2	1	4	15	**0.00568**
	%	-	1.71	2.90	1.53	2.27	15.38	2.83	

NCBR Narodowe Centrum Badań i Rozwoju

„Nowej generacji środki regulujące apetyt – użyteczne w opiece długoterminowej oraz kontroli masy ciała" Akronim RANG.e
Projekt finansowany przez Narodowe Centrum Badań i Rozwoju
Numer umowy: LIDER/48/0191/L-11/19/NCBR/2020

LIDER

Appetite increasesfor spicy products	*n*	-	**11**	**33**	**16**	6	6	68	0.48773
	%	-	**9.40**	**15.94**	**12.21**	13.21	13.64	12.83	
Appetite increasesfor flour products	*n*	8	**24**	**26**	**19**	10	5	86	0.18389
	%	40.00	**20.51**	**12.56**	**14.50**	22.73	19.23	16.23	
It increasescravings for poultry and/or meat	*n*	1	**26**	**28**	**18**	4	6	83	0.18698
	%	20.00	**22.22**	**13.53**	**13.74**	9.09	23.08	15.66	
It increasescraving for dairy products	*n*	-	4	3	-	1	2	10	0.10584
	%	-	40.00	1.45	-	2.27	7.69	1.89	
Croquet	*n*	-	-	5	2	1	2	10	0.18478
	%	-	-	2.42	1.53	2.27	7.69	1.89	
Borscht	*n*	-	-	1	7	2	1	11	0.01711
	%	-	-	0.48	5.34	4.55	3.85	2.08	
N/A (no increase/decrease in appetite)	*n*	1	**33**	**82**	**67**	**18**	**11**	212	**0.01283**
	%	20.00	**28.21**	**39.61**	**51.15**	**40.91**	**42.31**	40.00	

p - probability in the statistical chi2 test (significance level $\alpha < 0.05$), statistically significant differences in bold

Question 34.
The scents of which products make you feel safe?

Table 34. Smells causing a sense of security and the weight-height index of the surveyed people (n=530)

		Underweight	*Correct body weight*	*Overweight*	*I degree obesity*	*Grade II obesity*	*Grade III obesity*	*Total*	*p*
Coffee	*n*	3	**50**	**91**	**57**	**22**	**14**	237	0.82710
	%	30.00	**42.74**	**43.96**	**43.51**	**50.00**	**53.85**	44.72	
Black tea	*n*	1	**13**	**18**	**14**	2	2	50	0.74086
	%	20.00	**11.11**	**8.70**	**10.69**	4.55	7.69	9.43	
Dark chocolate	*n*	-	**13**	**23**	**13**	8	3	60	0.70437
	%	-	**11.11**	**11.11**	**9.92**	18.18	11.54	11.32	
Cheese	*n*	1	5	**12**	**14**	4	3	39	0.25759
	%	20.00	4.27	**5.80**	**10.69**	9.09	11.54	7.36	
Blue cheese	*n*	-	10	**11**	7	1	4	33	0.23642
	%	-	8.55	**5.31**	5.34	2.27	15.38	6.23	
Cooked egg	*n*	-	48	23	14	4	2	91	**0.00000**
	%	-	41.03	11.11	10.69	9.09	7.69	17.17	

NCBR Narodowe Centrum Badań i Rozwoju

„Nowej generacji środki regulujące apetyt – użyteczne w opiece długoterminowej oraz kontroli masy ciała" Akronim RANG.e
Projekt finansowany przez Narodowe Centrum Badań i Rozwoju
Numer umowy: LIDER/48/0191/L-11/19/NCBR/2020

LIDER

Fried salmon	*n*	-	9	10	6	5	4	34	0.18350
	%	-	7.69	4.83	4.58	11.36	15.38	6.42	
Oatmeal in milk	*n*	-	**53**	**17**	**15**	6	5	96	**0.00000**
	%	-	**45.30**	**8.21**	**11.45**	13.64	19.23	18.11	
Homogenized vanilla cheese	*n*	1	**13**	**19**	**13**	6	3	55	0.91827
	%	20.00	**11.11**	**9.18**	**9.92**	13.64	11.54	10.38	
Chocolate ice cream	*n*	-	9	**33**	**16**	5	1	64	0.19253
	%	-	7.69	**15.94**	**12.21**	11.36	3.85	12.08	
Vanilla ice cream	*n*	1	7	**37**	**21**	10	8	84	**0.00976**
	%	20.00	5.98	**17.87**	**16.03**	22.73	30.77	15.85	
Fruit sorbet	*n*	1	9	**12**	9	4	3	38	0.71881
	%	20.00	7.69	**5.80**	6.87	9.09	11.54	7.17	
I don't have that feeling after consuming any product	*n*	1	**16**	**53**	**33**	10	4	117	0.17136
	%	20.00	**13.68**	**25.60**	**25.19**	22.73	15.38	22.08	

p - probability in the statistical chi2 test (significance level $\alpha < 0.05$), statistically significant differences in bold

NCBR Narodowe Centrum Badań i Rozwoju

„Nowej generacji środki regulujące apetyt – użyteczne w opiece długoterminowej oraz kontroli masy ciała" Akronim RANG.e
Projekt finansowany przez Narodowe Centrum Badań i Rozwoju
Numer umowy: LIDER/48/0191/L-11/19/NCBR/2020

LIDER

Question 35.
The scents of which products make you feel blissful?

Table 35. Smells causing a sense of bliss and the weight-height index of the subjects (n=530)

		Underweight	*Correct body weight*	*Overweight*	*I degree obesity*	*Grade II obesity*	*Grade III obesity*	*Total*	*p*
Coffee	*n*	1	**69**	**84**	**45**	**17**	10	226	**0.00257**
	%	20.00	**58.97**	**40.58**	**34.35**	**39.64**	38.46	42.64	
Black tea	*n*	-	4	**11**	8	1	2	36	0.79471
	%	-	3.42	**5.31**	6.11	2.27	7.69	4.91	
Dark chocolate	*n*	1	**21**	**37**	**27**	10	7	103	0.87089
	%	20.00	**17.95**	**17.87**	**20.61**	22.73	36.92	19.43	
Cheese	*n*	1	2	**12**	8	1	2	26	0.23214
	%	20.00	1.71	**5.80**	6.11	2.27	7.69	4.91	
Blue cheese	*n*	1	4	**12**	4	2	4	27	0.07414
	%	20.00	3.42	**5.80**	3.05	4.55	15.38	5.09	
Cooked egg	*n*	-	**14**	9	6	3	2	34	0.12440
	%	-	**11.97**	4.35	4.58	6.82	7.69	6.42	

NCBR Narodowe Centrum Badań i Rozwoju

„Nowej generacji środki regulujące apetyt – użyteczne w opiece długoterminowej oraz kontroli masy ciała" Akronim RANG.e
Projekt finansowany przez Narodowe Centrum Badań i Rozwoju
Numer umowy: LIDER/48/0191/L-11/19/NCBR/2020

LIDER

Fried salmon	*n*	-	**13**	**15**	9	5	6	48	0.10244
	%	-	**11.11**	**7.25**	6.87	11.36	23.08	9.06	
Oatmeal in milk	*n*	1	**24**	**13**	10	5	3	56	**0.00296**
	%	20.00	**20.51**	**6.82**	7.63	11.36	11.54	10.57	
Homogenized vanilla cheese	*n*	2	**40**	**23**	**21**	7	6	99	**0.00002**
	%	40.00	**34.19**	**11.11**	**16.03**	15.91	23.08	18.68	
Chocolate ice cream	*n*	1	**43**	**57**	**26**	9	8	144	0.06728
	%	20.00	**36.75**	**27.54**	**19.85**	20.45	30.77	27.17	
Vanilla ice cream	*n*	2	**45**	**48**	**38**	**17**	8	158	0.06318
	%	40.00	**38.46**	**23.19**	**29.01**	**38.64**	30.77	29.81	
Fruit sorbet	*n*	1	**11**	**16**	10	7	3	48	0.51937
	%	20.00	**9.40**	**7.73**	7.63	15.91	11.54	9.06	
I don't have that feeling after consuming any product	*n*	1	**11**	**36**	**22**	5	2	77	0.32229
	%	20.00	**9.40**	**17.39**	**16.79**	11.36	7.69	14.53	

p - probability in the statistical chi2 test (significance level α < 0.05), statistically significant differences in bold

NCBR Narodowe Centrum Badań i Rozwoju

„Nowej generacji środki regulujące apetyt – użyteczne w opiece długoterminowej oraz kontroli masy ciała" Akronim RANG.e
Projekt finansowany przez Narodowe Centrum Badań i Rozwoju
Numer umowy: LIDER/48/0191/L-11/19/NCBR/2020

LIDER

Question 36.
Please select two of the following dishes that are the most appetizing for you.

Table 36. The most appetizing dishes according to the respondents, and their weight-height index (n=530)

		Underweight	*Correct body weight*	*Overweight*	*I degree obesity*	*Grade II obesity*	*Grade III obesity*	*Total*	*p*
vegetable salad	*n*	2	**21**	**63**	**27**	9	4	126	0.07564
	%	40.00	**17.95**	**30.43**	**20.61**	20.45	15.38	23.77	
Beef stew with buckwheat	*n*	-	**29**	**31**	**28**	**12**	6	106	0.15880
	%	-	**24.79**	**14.98**	**21.37**	**27.27**	23.08	20.00	
Letcho with chicken and white rice	*n*	1	8	**29**	**20**	8	7	73	0.08535
	%	20.00	6.84	**14.01**	**15.27**	18.18	26.92	13.77	
Cucumber soup	*n*	3	**54**	**93**	**48**	**17**	7	222	0.26932
	%	60.00	**46.15**	**44.93**	**36.64**	**38.64**	26.92	41.89	
Tomato soup with noodles	*n*	1	**29**	**53**	**31**	10	3	127	0.75549
	%	20.00	**24.79**	**25.60**	**23.66**	22.73	11.54	23.96	
Pancakes with white cheese and jam	*n*	3	**23**	**53**	**34**	**12**	5	130	0.33194
	%	60.00	**19.66**	**25.60**	**25.95**	**27.27**	19.23	24.53	

NCBR Narodowe Centrum Badań i Rozwoju

„Nowej generacji środki regulujące apetyt – użyteczne w opiece długoterminowej oraz kontroli masy ciała" Akronim RANG.e
Projekt finansowany przez Narodowe Centrum Badań i Rozwoju
Numer umowy: LIDER/48/0191/L-11/19/NCBR/2020

LIDER

Dumplings	*n*	-	**37**	**52**	**34**	**14**	10	147	0.34996
	%	-	**31.62**	**25.12**	**25.95**	**31.82**	38.46	27.74	
Croquette with cabbage and mushrooms	*n*	1	**28**	**43**	**27**	8	8	115	0.83132
	%	20.00	**23.93**	**20.77**	**20.61**	18.18	30.77	21.70	
Cod fried in butter with garlic and dill	*n*	1	**21**	**56**	**29**	5	4	116	p=.1675 4
	%	20.00	**17.95**	**27.05**	**22.14**	11.36	15.38	21.89	

p - probability in the statistical chi2 test (significance level $\alpha < 0.05$), statistically significant differences in bold

Question 37.
Please select two of the following dishes that are the least appetizing for you.

Table 37. The least appetizing dishes according to the respondents, and their weight-height index (n=530)

		Underw eight	*Correct body weight*	*Overwei ght*	*I degree obesity*	*Grade II obesity*	*Grade III obesity*	*Total*	*p*
vegetable salad	*n*	2	**38**	**41**	**26**	**11**	10	128	0.04238
	%	40.00	**32.48**	**19.81**	**19.85**	**25.00**	38.46	24.15	
Beef stew with buckwheat	*n*	2	**26**	**61**	**39**	**14**	6	148	0.64550
	%	40.00	**22.22**	**29.47**	**29.77**	**31.82**	23.08	27.92	
	n	1	**36**	**58**	**22**	**12**	8	137	0.15325

Letcho with chicken and white rice	%	20.00	**30.77**	**28.02**	**16.79**	**27.27**	30.77	25.85	
Cucumber soup	*n*	-	**24**	**37**	**26**	4	-	91	0.07252
	%	-	**20.51**	**17.87**	**19.85**	9.09	-	17.17	
Tomato soup with noodles	*n*	-	**15**	**24**	**19**	7	5	70	0.77028
	%	-	**12.82**	**11.59**	**14.50**	15.91	19.23	13.21	
Pancakes with white cheese and jam	*n*	-	**23**	**39**	**24**	5	9	100	0.21572
	%	-	**19.66**	**18.84**	**18.32**	11.36	34.62	18.87	
Dumplings	*n*	3	23	43	28	6	1	104	0.05767
	%	60.00	19.66	20.77	21.37	13.64	3.85	19.62	
Croquette with cabbage and mushrooms	*n*	2	**26**	**47**	**35**	**13**	5	128	0.75023
	%	40.00	**22.22**	**22.71**	**26.72**	**29.55**	19.23	24.15	
Cod fried in butter with garlic and dill	*n*	-	**17**	**52**	**32**	**12**	4	117	0.14588
	%	-	**14.53**	**25.12**	**24.43**	**27.27**	15.38	22.08	

p - probability in the statistical chi2 test (significance level $\alpha < 0.05$), statistically significant differences in bold

Question 38.
Please select two of the following products that are the most appetizing for you.

Table 38. The most appetizing products according to the respondents, and their weight-height index (n=530)

		Underweight	*Correct body weight*	*Overweight*	*I degree obesity*	*Grade II obesity*	*Grade III obesity*	*Total*	*p*
Chocolate ice cream	*n*	1	**31**	**82**	**38**	**12**	**12**	176	0.06705
	%	20.00	**26.50**	**39.61**	**29.01**	**27.27**	**46.15**	33.21	
Vanilla ice cream	*n*	3	**25**	**67**	**60**	**22**	8	185	**0.00036**
	%	60.00	**21.37**	**32.37**	**45.80**	**50.00**	30.77	34.91	
Bitter chocolate	*n*	-	**15**	**40**	**20**	6	4	85	0.58318
	%	-	**12.82**	**19.32**	**15.27**	13.64	15.38	16.04	
Vanilla/cream pudding	*n*	2	**34**	**45**	**26**	10	5	122	0.49736
	%	40.00	**29.06**	**21.74**	**19.85**	22.73	19.23	23.02	
Yeast cake with crumble	*n*	3	**63**	**78**	**49**	**14**	8	215	**0.02309**
	%	60.00	**53.85**	**37.68**	**37.40**	**31.82**	30.77	40.57	
Sweet bun with blueberries	*n*	-	**59**	**63**	**43**	**14**	**11**	190	**0.00357**
	%	-	**50.43**	**30.43**	**32.82**	**31.5**	**42.31**	35.85	

NCBR Narodowe Centrum Badań i Rozwoju

„Nowej generacji środki regulujące apetyt – użyteczne w opiece długoterminowej oraz kontroli masy ciała" Akronim RANG.e
Projekt finansowany przez Narodowe Centrum Badań i Rozwoju
Numer umowy: LIDER/48/0191/L-11/19/NCBR/2020

LIDER

Awning cookies	*n*	1	5	**25**	**16**	7	2	56	0.16722
	%	20.00	4.27	**12.08**	**12.21**	15.91	7.69	10.57	

p - probability in the statistical chi2 test (significance level α < 0.05), statistically significant differences in bold

Question 39.
Please select two of the following products that are the least appetizing for you.

Table 39. The least appetizing products according to the respondents, and their weight-height ratio (n=530)

		Underweight	*Correct body weight*	*Overweight*	*I degree obesity*	*Grade II obesity*	*Grade III obesity*	*Total*	*p*
Chocolate ice cream	*n*	1	**14**	**39**	**28**	9	6	97	0.47310
	%	20.00	**11.97**	**18.84**	**21.37**	20.45	23.08	6.30 p.m	
Vanilla ice cream	*n*	2	**20**	**25**	**20**	2	2	71	0.11583
	%	40.00	**17.09**	**12.08**	**15.27**	4.55	7.69	13.40	
Bitter chocolate	*n*	3	**60**	**72**	**54**	**17**	9	215	0.08417
	%	60.00	**51.28**	**34.78**	**41.22**	**38.64**	34.62	40.57	
Vanilla/cream pudding	*n*	-	**23**	**54**	**thirty**	**12**	6	125	0.60336
	%	-	**19.66**	**26.09**	**22.90**	**27.27**	23.08	23.58	
Yeast cake with crumble	*n*	-	**11**	**32**	**19**	10	8	80	0.05362

	%	-	**9.40**	**15.46**	**14.50**	22.73	30.77	15.09	
Sweet bun with blueberries	*n*	-	**15**	**54**	**26**	**12**	2	109	**0.02036**
	%	-	**12.82**	**20.09**	**19.85**	**27.27**	7.69	20.57	
Awning cookies	*n*	4	**86**	**123**	**75**	**19**	**13**	320	**0.00566**
	%	80.00	**73.50**	**59.42**	**57.25**	**43.18**	**50.00**	60.38	

p - probability in the statistical chi2 test (significance level α < 0.05), statistically significant differences in bold

Question 40.
Please select two of the following fruits that are the most appetizing for you.

Table 40. The most appetizing fruits according to the respondents, and their weight-height index (n=530)

		Underweight	*Correct body weight*	*Overweight*	*I degree obesity*	*Grade II obesity*	*Grade III obesity*	*Total*	*p*
Strawberries	*n*	2	**79**	**116**	**77**	**23**	**14**	311	0.29820
	%	40.00	**67.52**	**56.04**	**58.78**	**52.27**	**53.85**	58.68	
Watermelon	*n*	-	8	**44**	**29**	10	7	98	**0.00797**
	%	-	6.84	**21.26**	**22.14**	22.73	26.92	18.49	
Banana	*n*	1	9	**37**	**25**	6	2	80	0.10301
	%	20.00	7.69	**17.87**	**19.08**	13.64	7.69	15.09	

NCBR Narodowe Centrum Badań i Rozwoju

„Nowej generacji środki regulujące apetyt – użyteczne w opiece długoterminowej oraz kontroli masy ciała" Akronim RANG.e
Projekt finansowany przez Narodowe Centrum Badań i Rozwoju
Numer umowy: LIDER/48/0191/L-11/19/NCBR/2020

LIDER

Pineapple	*n*	1	5	**23**	**14**	2	7	52	**0.00981**
	%	20.00	4.27	**11.11**	**10.69**	4.55	26.92	9.81	
Peach	*n*	1	**29**	**42**	**25**	**11**	4	112	0.81075
	%	20.00	**24.79**	**20.29**	**19.08**	**25.00**	15.38	21.13	
Lemon	*n*	-	1	4	3	3	1	12	0.33785
	%	-	0.85	1.93	2.29	6.82	3.85	2.26	
Apple	*n*	3	**40**	**48**	**24**	8	4	127	**0.01277**
	%	60.00	**34.19**	**23.19**	**18.32**	18.18	15.38	23.96	
Pear	*n*	-	**23**	**33**	**19**	4	3	82	0.50855
	%	-	**19.66**	**15.94**	**14.50**	9.09	11.54	15.47	
grapes	*n*	1	**29**	**41**	**26**	**16**	3	116	0.13308
	%	20.00	**24.79**	**19.81**	**19.85**	**36.36**	11.54	21.89	
Orange	*n*	1	7	**22**	**15**	4	4	53	0.55979
	%	20.00	5.98	**10.63**	**11.45**	9.09	15.38	10.00	

p - probability in the statistical chi2 test (significance level $\alpha < 0.05$), statistically significant differences in bold

Question 41.
Please select two of the following fruits that are the least appetizing for you.

Table 41. The least appetizing fruits according to the respondents, and their weight-height index (n=530)

		Underweight	*Correct body weight*	*Overweight*	*I degree obesity*	*Grade II obesity*	*Grade III obesity*	*Total*	*p*
Strawberries	*n*	-	3	7	7	-	1	18	0.62342
	%	-	2.56	3.38	5.34	-	3.85	3.40	
Watermelon	*n*	3	**55**	**66**	**32**	8	3	167	**0.00008**
	%	60.00	**47.01**	**31.88**	**24.43**	1818	11.54	31.51	
Banana	*n*	1	**15**	**35**	**12**	4	4	71	0.38734
	%	20.00	**12.82**	**16.91**	**9.16**	9.09	15.38	13.40	
Pineapple	*n*	-	**59**	**66**	**40**	**14**	7	186	**0.00310**
	%	-	**50.43**	**31.88**	**30.53**	**31.82**	26.92	35.09	
Peach	*n*	-	9	**20**	**17**	3	1	50	0.52435
	%	-	7.69	**9.66**	**12.98**	6.82	3.85	9.43	
Lemon	*n*	3	**53**	**107**	**59**	**thirty**	**16**	268	0.07820
	%	60.00	**45.30**	**51.69**	**45.05**	**68.18**	**61.54**	50.57	

NCBR Narodowe Centrum Badań i Rozwoju

„Nowej generacji środki regulujące apetyt – użyteczne w opiece długoterminowej oraz kontroli masy ciała" Akronim RANG.e
Projekt finansowany przez Narodowe Centrum Badań i Rozwoju
Numer umowy: LIDER/48/0191/L-11/19/NCBR/2020

LIDER

Apple	*n*	1	3	**12**	**18**	6	6	46	**0.00085**
	%	20.00	2.56	**5.80**	**13.74**	13.64	23.08	8.68	
Pear	*n*	1	**13**	**thirty**	**24**	9	5	82	0.56967
	%	20.00	**11.11**	**14.49**	**18.32**	20.45	19.23	15.47	
grapes	*n*	-	**11**	**26**	**18**	8	4	67	0.64159
	%	-	**9.40**	**12.56**	**13.74**	18.18	15.38	12.64	
Orange	*n*	1	10	**36**	**22**	4	3	76	0.24464
	%	20.00	8.55	**17.39**	**16.79**	9.09	11.54	14.34	

p - probability in the statistical chi2 test (significance level $\alpha < 0.05$), statistically significant differences in bold

Question 42.
Please select two of the following fragrances that are the most appetizing for you.

Table 42. The most appetizing fragrances according to the respondents, and their weight-height index (n=530)

		Underweight	*Correct body weight*	*Overweight*	*I degree obesity*	*Grade II obesity*	*Grade III obesity*	*Total*	*p*
Coffee	*n*	3	**60**	**125**	**73**	**26**	**14**	301	0.72988
	%	60.00	**51.28**	**60.39**	**55.73**	**59.09**	**53.85**	56.79	
Black tea	*n*	-	9	**15**	8	2	6	40	0.06509

NCBR Narodowe Centrum Badań i Rozwoju

„Nowej generacji środki regulujące apetyt – użyteczne w opiece długoterminowej oraz kontroli masy ciała" Akronim RANG.e
Projekt finansowany przez Narodowe Centrum Badań i Rozwoju
Numer umowy: LIDER/48/0191/L-11/19/NCBR/2020

LIDER

	%	-	7.69	**7.25**	6.11	4.55	2.308	7.55	
Cinnamon	*n*	3	18	60	33	6	7	127	**0.01584**
	%	60.00	15.38	28.99	25.19	13.64	26.92	23.96	
Vanilla	*n*	2	**39**	**61**	**36**	**15**	5	158	0.68927
	%	40.00	**33.33**	**29.47**	**27.48**	**34.09**	19.23	29.81	
Rosemary	*n*	-	2	**12**	7	4	2	27	0.40548
	%	-	1.71	**5.80**	5.34	9.09	7.69	5.09	
Kefir	*n*	-	**11**	**18**	**11**	3	4	47	0.82568
	%	-	**9.40**	**8.70**	**8.40**	6.82	15.38	8.87	
Grilled sausage	*n*	-	**38**	**48**	**38**	**14**	4	142	0.17063
	%	-	**32.48**	**23.19**	**29.01**	**31.82**	15.38	26.79	
Roast	*n*	-	**23**	**18**	**12**	8	2	63	**0.02895**
	%	-	**19, 66**	**8.70**	**9.16**	18.18	7.69	11.89	
Naturally smoked bacon	*n*	-	**23**	**38**	**29**	7	5	102	0.80192
	%		**19.66**	**18.36**	**22.14**	15.91	19.23	19.25	
Maple syrup	*n*	2	5	2	4	1	-	14	**0.00001**

	%	40.00	4.27	09.7	3.05	2.27	-	2.64	
Cheese	*n*	-	6	**16**	5	2	2	31	0.69554
	%	-	5.13	**7.73**	3.82	4.55	7.69	5.85	

p - probability in the statistical chi2 test (significance level α < 0.05), statistically significant differences in bold

Question 43.
Please select two of the following fragrances that are the least appetizing for you.

Table 43. The least appetizing smells according to the respondents, and their weight-height index (n=530)

		Underweight	*Correct body weight*	*Overweight*	*I degree obesity*	*Grade II obesity*	*Grade III obesity*	*Total*	*p*
Coffee	*n*	-	2	8	10	6	1	27	0.82190
	%	-	7.69	6.84	4.83	4.58	2.27	5.09	
Black tea	*n*	1	**24**	**45**	**20**	**12**	6	108	0.58550
	%	20.00	**20.51**	**21.74**	**15.27**	**27.27**	23.08	20.38	
Cinnamon	*n*	-	**16**	**28**	**25**	6	8	83	0.16856
	%	-	**13.68**	**13.53**	**19.08**	13.64	30.77	15.66	
Vanilla	*n*	-	6	**11**	**11**	6	3	37	0.29501
	%	-	5.13	**5.31**	**8.40**	13.64	11.54	6.98	

NCBR Narodowe Centrum Badań i Rozwoju

„Nowej generacji środki regulujące apetyt – użyteczne w opiece długoterminowej oraz kontroli masy ciała" Akronim RANG.e
Projekt finansowany przez Narodowe Centrum Badań i Rozwoju
Numer umowy: LIDER/48/0191/L-11/19/NCBR/2020

LIDER

Rosemary	*n*	-	**45**	**61**	**32**	**16**	3	157	**0.02136**
	%	-	**38.46**	**29.47**	**24.43**	**36.36**	11.54	29.62	
Kefir	*n*	1	**22**	**52**	**37**	9	9	130	0.41995
	%	20.00	**18.18**	**25.12**	**28.24**	20.45	20.45	24.53	
Grilled sausage	*n*	3	**18**	**31**	**13**	5	4	74	**0.04636**
	%	60.00	**15.38**	**14.98**	**9.92**	11.36	15.38	13.96	
Roast	*n*	2	5	**26**	**20**	5	1	59	**0.01650**
	%	40.00	4.27	**12.56**	**15.27**	11.36	3.85	11.13	
Naturally smoked bacon	*n*	2	**14**	**31**	**21**	3	3	74	0.32108
	%	40.00	**11.97**	**14.98**	**16.03**	6.82	11.54	13.96	
Maple syrup	*n*	1	**49**	**80**	**44**	**16**	10	200	0.76162
	%	20.00	**41.88**	**38.65**	**33.59**	**36.36**	38.46	37.74	
Cheese	*n*	-	**27**	**31**	**23**	6	3	90	0.34460
	%	-	**23.08**	**14.98**	**17.56**	13.64	11.54	16.98	

p - probability in the statistical chi2 test (significance level $\alpha < 0.05$), statistically significant differences in bold

NCBR Narodowe Centrum Badań i Rozwoju

„Nowej generacji środki regulujące apetyt – użyteczne w opiece długoterminowej oraz kontroli masy ciała" Akronim RANG.e
Projekt finansowany przez Narodowe Centrum Badań i Rozwoju
Numer umowy: LIDER/48/0191/L-11/19/NCBR/2020

LIDER

Question 44.
Please select two of the following dishes that are the most filling for you.

Table 44. The most filling dishes according to the respondents, and their weight-height index (n=530)

		Underweight	*Correct body weight*	*Overweight*	*I degree obesity*	*Grade II obesity*	*Grade III obesity*	*Total*	*p*
vegetable salad	*n*	-	8	**21**	**16**	-	-	45	0.06377
	%	-	6.84	**10.14**	**12.21**	-	-	8.49	
Beef stew with buckwheat	*n*	2	**75**	**97**	**61**	**25**	**18**	278	**0.01344**
	%	40.00	**64.10**	**46.86**	**46.56**	**56.82**	**69.23**	52.45	
Letcho with chicken and white rice	*n*	3	**20**	**49**	**34**	10	6	122	0.23083
	%	60.00	**17.09**	**23.67**	**25.95**	22.73	23.08	23.02	
Cucumber soup	*n*	-	6	**12**	**11**	2	1	32	0.82212
	%	-	5.13	**5.80**	**8.40**	4.55	3.85	6.04	
Tomato soup with noodles	*n*	-	10	**thirty**	**15**	-	3	58	0.09308
	%	-	8.55	**14.49**	**11.45**	-	11.54	10.94	
Pancakes with white cheese and jam	*n*	4	**50**	55	33	**15**	1	158	**0.00007**
	%	80.00	**42.74**	**26.57**	**25.19**	**34.09**	3.85	29.81	

NCBR Narodowe Centrum Badań i Rozwoju

„Nowej generacji środki regulujące apetyt – użyteczne w opiece długoterminowej oraz kontroli masy ciała" Akronim RANG.e
Projekt finansowany przez Narodowe Centrum Badań i Rozwoju
Numer umowy: LIDER/48/0191/L-11/19/NCBR/2020

LIDER

Dumplings	*n*	-	**23**	**55**	**35**	**18**	**16**	147	**0.00017**
	%	-	**19.66**	**26.57**	**26.72**	**40.91**	**61.54**	27.74	
Croquette with cabbage and mushrooms	*n*	1	**26**	**53**	**31**	**12**	3	126	0.69640
	%	20.00	**22.22**	**25.06**	**23.66**	**27.27**	11.54	23.77	
Cod fried in butter with garlic and dill	*n*	-	**15**	**33**	**16**	2	4	70	0.38883
	%	-	**12.82**	**15.94**	**12.21**	4.55	15.38	13.21	

p - probability in the statistical chi2 test (significance level α < 0.05), statistically significant differences in bold

Question 45.
Please select two of the following dishes that are the least filling for you.

Table 45. The least satiating dishes according to the respondents, and their weight-height ratio (n=530)

		Underweight	*Correct body weight*	*Overweight*	*I degree obesity*	*Grade II obesity*	*Grade III obesity*	*Total*	*p*
vegetable salad	*n*	4	**69**	**126**	**75**	**34**	**16**	324	0.24877
	%	80.00	**58.97**	**60.87**	**57.25**	**77.27**	**61.54**	61.13	
Beef stew with buckwheat	*n*	-	5	9	5	2	3	24	0.64174
	%	-	4.27	4.35	3.82	4.55	11.54	4.53	
	n	-	10	**15**	**11**	5	3	44	0.89316

Letcho with chicken and white rice	%	-	8.55	**7.25**	**8.40**	11.36	11.54	8.30	
Cucumber soup	*n*	1	**71**	**112**	**60**	**20**	9	273	**0.03742**
	%	20.00	**60.68**	**54.11**	**45.80**	**45.45**	34.62	51.51	
Tomato soup with noodles	*n*	2	**38**	**38**	**34**	4	6	122	**0.01166**
	%	40.00	**32.48**	**18.36**	**25.95**	9.09	23.08	23.02	
Pancakes with white cheese and jam	*n*	1	10	**22**	**18**	6	5	62	0.57783
	%	20.00	8.55	**10.63**	**13.74**	13.64	19.23	11.70	
Dumplings	*n*	1	**11**	**17**	8	1	5	43	0.14338
	%	20.00	**9.40**	**8.21**	6.11	2.27	19.23	8.11	
Croquette with cabbage and mushrooms	*n*	-	9	**23**	**20**	1	4	57	0.13402
	%	-	7.69	**11.11**	**15.27**	2.27	15.38	10.75	
Cod fried in butter with garlic and dill	*n*	1	10	**37**	**18**	**11**	**-**	77	**0.01743**
	%	20.00	8.55	**17.87**	**13.74**	**25.00**	-	14.53	

p - probability in the statistical chi2 test (significance level $\alpha < 0.05$), statistically significant differences in bold

NCBR Narodowe Centrum Badań i Rozwoju

„Nowej generacji środki regulujące apetyt – użyteczne w opiece długoterminowej oraz kontroli masy ciała" Akronim RANG.e
Projekt finansowany przez Narodowe Centrum Badań i Rozwoju
Numer umowy: LIDER/48/0191/L-11/19/NCBR/2020

LIDER

Question 46.
Please select two of the following products that are the most filling for you.

Table 46. The most satiating products according to the respondents, and their weight-height ratio (n=530)

		Underweight	*Correct body weight*	*Overweight*	*I degree obesity*	*Grade II obesity*	*Grade III obesity*	*Total*	*p*
Chocolate ice cream	*n*	-	**14**	**55**	**35**	9	2	115	**0.00749**
	%	-	**11.97**	**26.57**	**26.72**	20.45	7.69	21.07	
Vanilla ice cream	*n*	-	8	**thirty**	**18**	6	5	67	0.29287
	%	-	6.84	**14.49**	**13.74**	13.64	19.23	12.64	
Bitter chocolate	*n*	1	**11**	**23**	**14**	5	7	61	0.21699
	%	20.00	**9.40**	**11.11**	**10.69**	11.36	26.92	11.51	
Vanilla/cream pudding	*n*	2	**33**	**58**	**43**	**12**	8	156	0.92436
	%	40.00	**28.21**	**28.02**	**32.82**	**27.27**	30.77	29.43	
Yeast cake with crumble	*n*	5	**88**	**125**	**79**	**28**	**13**	338	**0.02061**
	%	100.00	**75.21**	**60.39**	**60.31**	**63.64**	**50.00**	63.77	
Sweet bun with blueberries	*n*	2	**72**	**97**	**60**	**22**	**11**	264	0.11822
	%	40.00	**61.54**	**46.86**	**45.80**	**50.00**	**42.31**	49.81	

Awning cookies	*n*	-	7	**17**	4	4	4	36	0.19014
	%	-	5.98	**8.21**	3.05	9.069	15.38	6.79	

p - probability in the statistical chi2 test (significance level α < 0.05), statistically significant differences in bold

Question 47.
Please select two of the following products that are the least satiating for you.

Table 47. The least satiating products according to respondents, and their weight-height ratio (n=530)

		Underweight	*Correct body weight*	*Overweight*	*I degree obesity*	*Grade II obesity*	*Grade III obesity*	*Total*	*p*
Chocolate ice cream	*n*	3	**62**	**51**	**45**	**14**	7	182	**0.00002**
	%	60.00	**52.99**	**24.64**	**34.35**	**31.82**	26.92	34.34	
Vanilla ice cream	*n*	3	**69**	**87**	**58**	**20**	**11**	248	0.08154
	%	60.00	**58.97**	**42.03**	**44.27**	**45.45**	**42.31**	46.79	
Bitter chocolate	*n*	2	**26**	**75**	**39**	**14**	8	164	0.20858
	%	40.00	**22.22**	**26.23**	**29.77**	**31.82**	30.77	30.94	
Vanilla/cream pudding	*n*	-	**27**	**48**	**22**	5	2	104	0.12216
	%	-	**23.08**	**23.19**	**16.79**	11.36	7.69	19.62	
Yeast cake with crumble	*n*	-	5	**15**	**12**	5	2	39	0.59368

	%	-	4.27	**7.25**	**9.16**	11.36	7.69	7.36	
Sweet bun with blueberries	*n*	-	9	**23**	**18**	5	7	62	0.11241
	%	-	7.69	**11.11**	**13.74**	11.36	26.92	11.70	
Awning cookies	*n*	2	**36**	**95**	**53**	**23**	**14**	223	0.05382
	%	40.00	**30.77**	**45.89**	**40.46**	**52.27**	**53.85**	42.08	

p - probability in the statistical chi2 test (significance level α < 0.05), statistically significant differences in bold

Question 48.
Please select two of the following fruits that are the most filling for you.

Table 48. The most filling fruits according to the respondents, and their weight-height index (n=530)

		Underweight	*Correct body weight*	*Overweight*	*I degree obesity*	*Grade II obesity*	*Grade III obesity*	*Total*	*p*
Strawberries	*n*	1	6	**21**	**18**	4	4	54	0.26644
	%	20.00	5.13	**10.14**	**13.74**	9.09	15.38	10.19	
Watermelon	*n*	1	6	**19**	**13**	5	7	51	**0.02856**
	%	20.00	5.13	**9.18**	**9.92**	11.36	26.92	9.62	
Banana	*n*	2	**79**	**141**	**81**	**33**	**14**	350	0.27303
	%	40.00	**67.52**	**68.12**	**61.83**	**75.00**	**53.85**	66.04	

Pineapple	*n*	-	6	**14**	9	3	6	38	**0.04980**
	%	-	5.13	**6.76**	6.87	6.87	23.08	7.17	
Peach	*n*	-	**22**	**thirty**	**22**	8	4	89	0.82572
	%	-	**18.18**	**14.49**	**16.79**	18.18	15.38	16.23	
Lemon	*n*	-	-	1	1	-	-	2	0.93410
	%	-	-	0.48	0.76	-	-	0.38	
Apple	*n*	4	**60**	**60**	**54**	**17**	7	202	**0.00064**
	%	80.00	**51.28**	**28.99**	**41.22**	**38.64**	26.92	38.11	
Pear	*n*	2	**34**	**66**	**32**	7	7	148	0.30479
	%	40.00	**29.06**	**31.88**	**24.43**	15.91	26.92	27.92	
grapes	*n*	-	**12**	**42**	**18**	8	3	83	0.16843
	%	-	**10.26**	**20.29**	**13.74**	18.18	11.54	15.66	
Orange	*n*	-	8	**15**	6	4	-	33	0.58914
	%	-	6.84	**7.25**	4.58	9.09	-	6.23	

p - probability in the statistical chi2 test (significance level $\alpha < 0.05$), statistically significant differences in bold

Question 49.
Please select two of the following fruits that are the least satiating for you.

Table 49. The least satiating fruits according to the respondents, and their weight-height index (n=530)

		Underweight	*Correct body weight*	*Overweight*	*I degree obesity*	*Grade II obesity*	*Grade III obesity*	*Total*	*p*
Strawberries	*n*	2	**54**	**65**	**39**	**15**	8	183	0.09294
	%	40.00	**46.15**	**31.40**	**29.77**	**34.09**	30.77	34.53	
Watermelon	*n*	1	**81**	**124**	**78**	**29**	7	320	**0.00124**
	%	20.00	**69.23**	**59.90**	**59.54**	**65.91**	26.92	60.38	
Banana	*n*	-	6	9	5	1	3	24	0.55277
	%	-	5.13	4.35	3.82	2.27	11.54	4.53	
Pineapple	*n*	-	**21**	**17**	**14**	5	4	61	0.15113
	%	-	**17.95**	**8.21**	**20.69**	11.36	15.38	11.51	
Peach	*n*	-	-	**12**	10	3	3	28	0.05989
	%	-	-	**5.80**	7.63	6.82	11.54	5.28	
Lemon	*n*	3	**34**	**86**	**51**	**24**	**15**	213	**0.01453**
	%	60.00	**29.06**	**41.55**	**38.93**	**54.55**	**57.69**	40.19	

Apple	*n*	-	**13**	**32**	**14**	2	2	63	0.29241
	%	-	**11.11**	**15.46**	**10.69**	4.55	7.69	11.89	
Pear	*n*	-	1	9	7	1	2	20	0.37006
	%	-	0.85	4.35	5.34	2.27	7.69	3.77	
grapes	*n*	4	18	38	24	6	6	96	**0.01254**
	%	80.00	15.38	18.36	18.32	13.64	23.08	18.11	
Orange	*n*	-	2	10	10	2	2	26	0.37384
	%	-	1.71	4.83	7.63	4.55	7.69	4.91	

p - probability in the statistical chi2 test (significance level $\alpha < 0.05$), statistically significant differences in bold

Question 50.
Please select two of the following fragrances that are the most filling for you.

Table 50. The most satiating fragrances according to the respondents, and their weight-height index (n=530)

		Underweight	*Correct body weight*	*Overweight*	*I degree obesity*	*Grade II obesity*	*Grade III obesity*	*Total*	*p*
Coffee	*n*	1	**21**	**62**	**34**	9	5	132	0.22811
	%	20.00	**17.95**	**29.95**	**25.95**	20.45	19.23	24.91	
Black tea	*n*	1	4	3	8	1	3	20	**0.02072**

	%	20.00	3.42	1.45	6.11	2.27	11.54	3.77	
Cinnamon	*n*	-	4	**17**	**12**	4	4	41	0.29125
	%	-	3.42	**8.21**	**9.16**	9.09	15.38	7.74	
Vanilla	*n*	2	**14**	**27**	**12**	7	3	65	0.36567
	%	40.00	**11.97**	**13.04**	**9.16**	15.91	11.54	12.26	
Rosemary	*n*	-	1	1	2	1	1	6	0.65055
	%	-	0.85	0.48	1.53	2.27	3.85	1.13	
Kefir	*n*	-	2	**17**	9	4	6	38	**0.00653**
	%	-	1.71	**8.21**	6.87	9.09	23.08	7.17	
Grilled sausage	*n*	1	**78**	**111**	**67**	**24**	**16**	297	0.07182
	%	20.00	**66.67**	**53.62**	**51.15**	**54.55**	**61.54**	56.04	
Roast	*n*	2	**67**	**70**	**42**	**16**	5	202	**0.00010**
	%	40.00	**57.26**	**33.82**	**32.06**	**36.36**	19.23	38.11	
Naturally smoked bacon	*n*	2	**28**	**51**	**45**	**16**	5	157	0.32729
	%	40.00	**23.93**	**29.47**	**34.65**	**36.36**	19.23	29.62	
Maple syrup	*n*	-	2	**11**	4	3	2	22	0.45666

	%	-	1.71	**5.31**	3.05	6.82	7.69	4.15	
Cheese	*n*	1	10	**27**	**17**	4	-	59	0.32157
	%	20.00	8.55	**13.04**	**12.98**	9.09	-	11.13	

p - probability in the statistical chi2 test (significance level α < 0.05), statistically significant differences in bold

Question 51.
Please select two of the following fragrances that are the least satiating for you.

Table 51. The least satiating fragrances according to the respondents, and their weight-increase ratio (n=530)

		Underweight	*Correct body weight*	*Overweight*	*I degree obesity*	*Grade II obesity*	*Grade III obesity*	*Total*	*p*
Coffee	*n*	-	8	**27**	**21**	6	7	69	0.06979
	%	-	6.84	**13.04**	**16.03**	13.64	26.92	13.02	
Black tea	*n*	1	**49**	**103**	**46**	**18**	8	225	0.07658
	%	20.00	**41.88**	**49.76**	**35.11**	**40.91**	30.77	42.45	
Cinnamon	*n*	-	**19**	**36**	**20**	**11**	5	91	0.63510
	%	-	**16.24**	**17.39**	**15.27**	**25.00**	19.23	17.17	
Vanilla	*n*	2	17	thirty	16	9	4	78	0.49673
	%	40.00	14.53	14.49	12.21	20.45	15.38	14.72	

Rosemary	*n*	2	**46**	**47**	**23**	8	5	131	**0.00154**
	%	40.00	**39.32**	**22.71**	**17.65**	18.18	19.23	24.72	
Kefir	*n*	3	**30**	**69**	**51**	**18**	7	178	0.14899
	%	60.00	**25.64**	**33.33**	**38.93**	**40.91**	26.92	33.58	
Grilled sausage	*n*	-	5	**15**	6	1	2	29	0.67005
	%	-	4.27	**7.25**	4.58	2.27	7.69	5.47	
Roast	*n*	-	2	8	5	-	-	15	0.55112
	%	-	1.71	3.86	3.82	-	-	2.83	
Naturally smoked bacon	*n*	-	3	6	8	3	1	21	0.55660
	%	-	2.56	2.90	6.11	6.82	3.85	3.96	
Maple syrup	*n*	1	40	41	thirty	11	8	131	0.10407
	%	20.00	34.19	19.81	22.90	25.00	30.77	24.72	
Cheese	*n*	1	**12**	**24**	**23**	3	1	64	0.20500
	%	20.00	**10.26**	**11.59**	**17.56**	6.82	3.85	12.08	

p - probability in the statistical chi2 test (significance level $\alpha < 0.05$), statistically significant differences in bold

Question 52.
Is there any smell that would make you feel full faster after eating a meal? If yes, please indicate which one by entering the answer in the "Other" field.

Table 52. Smells causing the feeling of satiety in respondents, and their weight-growth index (n=530)

		Underweight	*Correct body weight*	*Overweight*	*I degree obesity*	*Grade II obesity*	*Grade III obesity*	*Total*	*p*
NO	*n*	5	**112**	**191**	**122**	**42**	**25**	497	0.78320
	%	100.00	**95.73**	**92.27**	**93.13**	**95.45**	**96.15**	93.77	
Smell of meat/poultry after frying/baking/smoking	*n*	-	2	5	4	-	-	11	0.79133
	%	-	1.71	2.42	3.05	-	-	2.08	
Smell of grilled products (sausage/neck)	*n*	-	1	3	3	-	1	8	0.76360
	%	-	0.85	1.45	2.29	-	3.85	1.51	
Coffee	*n*	-	1	2	-	1	-	4	0.74232
	%	-	0.85	0.97	-	2.27	-	0.75	
Spices (curry, cinnamon, garlic, pepper)	*n*	-	1	5	2		-	9	0.87837
	%	-	0.85	2.42	1.53	2.27	-	1.70	
An avocado	*n*	-	2	2	-	1	-	5	0.68293
	%	-	1.71	0.97	-	2.27	-	0.94	

Cooked egg	*n*	-	1	1	-	-	-	2	0.90711
	%	-	0.85	0.48	-	-	-	0.38	

p - probability in the statistical chi2 test (significance level α < 0.05), statistically significant differences in bold

Question 53.
Is there a scent that would make you feel hungry? If yes, please indicate which one by entering the answer in the "Other" field.

Table 55. Smells causing the feeling of hunger in respondents, and their weight-height index (n=530)

		Underweight	*Correct body weight*	*Overweight*	*I degree obesity*	*Grade II obesity*	*Grade III obesity*	*Total*	*p*
NO	*n*	3	**82**	**173**	**108**	**27**	**18**	420	**0.03437**
	%	60.00	**70.09,**	**83.57**	**81.82**	**61.36**	**69.23**	79.25	
Smell of meat/poultry/sausage after baking/frying/grilling	*n*	-	**20**	**17**	**13**	4	7	61	**0.02261**
	%	-	**17.09**	**8.21**	**9.92**	9.09	26.92	11.51	
The smell of bread (especially fresh)	*n*	1	10	**11**	5	4	1	32	0.38154
	%	20.00	8.55	**5.31**	3.82	9.09	3.85	6.04	
The smell of cake and sweet products	*n*	1	-	6	3	-	-	10	**0.01751**
	%	20.00	-	2.90	2.29	-	-	1.89	
Fast food products	*n*	-	6	4	2	1	-	13	0.41717

	%	-	5.13	1.93	1.53	2.27	-	2.45	

p - probability in the statistical chi2 test (significance level α < 0.05), statistically significant differences in bold
